# A General Framework for the Multiple Nonparametric Behrens–Fisher Problem With Dependent Replicates

**DOI:** 10.1002/sim.10262

**Published:** 2024-11-11

**Authors:** Erin Sprünken, Robert Mertens, Frank Konietschke

**Affiliations:** ^1^ Institute of Biometry and Clinical Epidemiology Charité ‐ Universitätsmedizin Berlin Berlin Germany; ^2^ Department of Neurosurgery Charité ‐ Universitätsmedizin Berlin Berlin Germany

**Keywords:** Behrens–Fisher, cluster, multiple testing, nonparametric, replicates

## Abstract

In many trials and experiments, subjects are not only observed once but multiple times, resulting in a cluster of possibly correlated observations (e.g., brain regions per patient). Observations often do not fulfill model assumptions of mixed models and require the use of nonparametric methods. In this article, we develop and present a purely nonparametric rank‐based procedure that flexibly allows the unbiased and consistent estimation of the Wilcoxon–Mann–Whitney effect $P(X<Y)+\frac{1}{2}P(X=Y)$ in clustered data designs. Compared with existing methods, we allow flexible weights to be used in effect estimation. Additionally, we develop global and multiple contrast test procedures to test null hypotheses formulated regarding the generalized Mann–Whitney effects and for the computation of range‐preserving simultaneous confidence intervals in a unified way. Extensive simulation studies show that these methods control the type‐I error rate well and have reasonable power to detect alternatives in various situations.

## 1 Introduction

In many trials and experiments, subjects are observed not just once under a specific condition but multiple times. This leads to several potentially *correlated replicates* of a particular outcome. For instance, dental experiments involving multiple teeth belonging to the same individual, pre‐clinical trials with animals housed together in the same cage, or social experiments where students from the same class or school are observed are common examples of such situations. The replicates are often called *clustered data* in the literature, facing challenges in making statistical inferences, mainly due to their rather involved dependency structure. Furthermore, their scale (e.g., in the case of ordinal data) and distributional shape (e.g., skewed distributions) often imply that means (expectations) are inappropriate quantities to use in the definition of treatment effects. Therefore, estimating a treatment effect and testing null hypotheses within a linear mixed model framework [1, 2, 3] assuming normally distributed random effects and errors are unsuitable in such situations. Semi‐parametric mixed‐models relaxing restrictive model assumptions and rank‐based estimators of effects in linear models are well established in statistical sciences [4, 5, 6, 7]. However, they usually assume data scales at least being metric or even continuous and hence cannot be used in the case of discrete or ordinal data [4]. This manuscript aims to overcome these issues and propose a purely nonparametric statistical inference framework for data analysis with (possibly) dependent replicates in general factorial designs in a unified way.

In recent years, several researchers developed purely nonparametric (rank‐based) statistical inference methods for analyzing clustered data in two independent and dependent samples [8, 9, 10, 11]. In principle, these methods are generalized Wilcoxon–Mann–Whitney or Wilcoxon signed rank tests, which can be used for testing the equality of the distribution functions [12, 13]. However, this formulation of the null hypothesis is rather strict, and neither allows for variance (or any other higher moments) heteroscedasticity nor the computation of confidence intervals. Therefore, test procedures and confidence intervals for the Mann–Whitney effect

(1) $$\begin{array}{ll} p & =P(X<Y)+\frac{1}{2}P(X=Y)\\ & \text{(X and Y are independent random variables)} \end{array}$$

have been proposed for two independent samples [14, 15, 16, 17], for partially complete clustered data [17] and for factorial pre‐post designs with clustered data [18]. Note that p is also known as *relative effect*, *probabilistic index* or *stress‐strength index* in the literature [19, 20, 21]. Rubarth et al. (2022) extended these procedures by devising global and multiple contrast tests for testing null hypotheses formulated in relative effects within factorial repeated measurement designs, accounting for potential missing values [22]. However, their work is limited to unweighted and weighted effect estimators, even though different weightings of observations within a cluster—such as weighting based on cluster size or the reliability of individual measurements—are of practical interest in many clustered data designs. Rubarth et al.'s procedures also require moderate to large sample sizes, which may not be feasible in all research settings.

This article aims to overcome these limitations and to provide (1) flexible, unbiased, and consistent point estimators of the relative effect, allowing for arbitrary cluster weightings. We derive the estimators' asymptotic multivariate normal distribution and consistent variance‐covariance matrix estimators that are consistent under very mild assumptions, making them applicable in most situations in statistical practice. We furthermore (2) propose Multiple Contrast Test Procedures (MCTPs), which do not only allow for making global but also local inferences and the computation of range‐preserving simultaneous confidence intervals (SCI). We approximate the distributions of their test statistics for small sample sizes with multivariate t distributions. Extensive simulation studies show that they control the nominal type‐I error rate well and have acceptable power to detect selected alternatives.

The remainder of the article is structured as follows: Section 2 motivates our work with a real data example, Section 3 introduces the statistical model and effects, and Section 4 deals with the derivation of point estimators, asymptotic properties, and the covariance matrix. Section 5 discusses different testing procedures, and Section 6 special cases of our model. Finally, Section 7 provides simulation results of the proposed methods, and in Section 8, we evaluate the real data example from Section 2. Eventually, Section 9 concludes our work.

## 2 Motivating Example

The motivating example arises from the discipline of neurosurgery. Moyamoya disease (MMD) is a rare cerebral angiopathy of unknown etiology, characterized by progressive spontaneous bilateral stenosis of the terminal internal carotid arteries (ICA) with compensatory formation of capillary collaterals resembling a “puff of smoke” (Japanese: Moyamoya) on cerebral angiography and treated by surgical revascularization [23]. Czabanka et al. proposed a new grading system for MMD combining functional and morphological parameters in 2011, also known as the “Berlin Grading System,” an ordinal scale ranging from one to six. Here, risk assessment is performed based on MRI findings, cerebrovascular reserve capacity (CVRC), and collateralization patterns in digital subtraction angiography (DSA) [24]. Each hemisphere is assessed separately depending on points for each category: DSA, MRI, and CVRC (see Table 1).

**TABLE 1:** The Berlin Grading System of Moyamoya Disease, adapted for evaluation by ${[}^{15}O]H_{2}O$ PET.

Variable	Characteristics	Points
DSA	Steno‐occlusive lesion + Moyamoya vessels	1
	Steno‐occlusive lesion + Moyamoya vessels + intracranial compensation routes	2
	Steno‐occlusive lesion + extra‐intracranial compensation routes	3
MRI	No Signs of ischemia/hemorrhage/atrophy	0
	Signs of ischemia/hemorrhage/atrophy	1
CVRC	Normal CVRC	0
	Reduced CVRC	2

*Note*: 1–2 points = Grade 1 (mild form), 3–4 points = Grade 2 (moderate form), 5–6 points = Grade 3 (severe form). Adapted from Czabanka et al. [24].

DSA was divided into three stages. One point was assigned to the presence of stenotic or occlusive lesions combined with typical Moyamoya vessels without intracranial or extra‐intracranial compensation routes. The combined appearance of these alterations and additional intracranial collateral pathways, such as leptomeningeal and/or pericallosal anastomosis, received two points. The presence of stenotic or occlusive lesions paralleled by the presence of extra‐intracranial collaterals was assigned three points. MRI was divided into two stages. No sign of ischemia/hemorrhage/atrophy was assigned zero points. Ischemic lesions, intracerebral hemorrhage, and atrophy were assigned one point. The CVRC results were defined as the third variable and were divided into two stages: normal or impaired. In the qualitative analysis, no increase of relative CVRC after acetazolamide was assigned two points, whereas the physiological increase of relative CVRC after acetazolamide was assigned zero points. By summarizing the numerical values of each variable per hemisphere, a total score with three grades of MMD/MMS was determined: 1–2 points were defined as Grade I (mild form), 3–4 points were defined as Grade II (moderate form) and 5–6 points were defined as Grade III (severe form). The grading system assesses each hemisphere separately; therefore, patients may have different grades per hemisphere.

**FIGURE 1:**
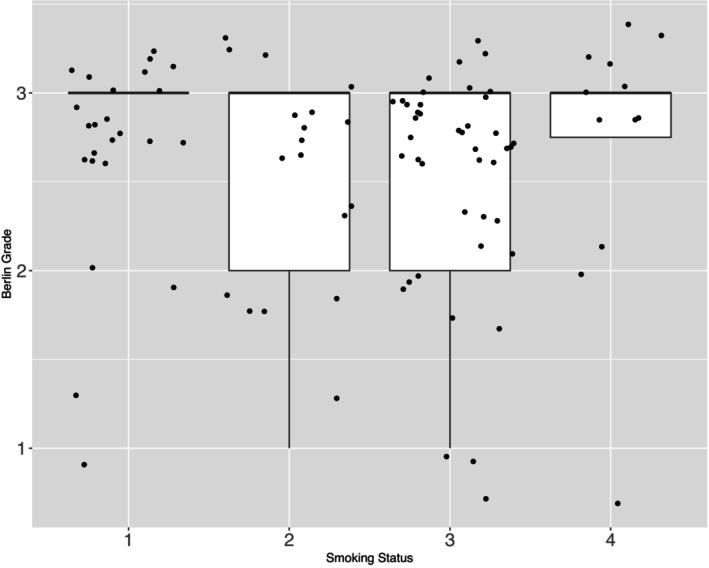
Boxplot of Berlin grades divided into the four smoking categories, clustering ignored.

A relevant research question is how smoking affects the severity of MMD. The variable smoking is represented as a categorical one, dividing the cohort (n=48) into four groups (1 = active smoker (n=12), 2 = former smoker (n=9), 3 = non‐smoker (n=21), 4 = unknown (n=6)). In this data, the status *unknown* is synonymous to *missing*. Since patients answer the question for the smoking status themselves, missing values may not occur randomly but rather systematically, and hence, missing values should be included as a smoking status in statistical inference. The trial aims to infer differences in the Berlin grade between these four groups. However, an issue is that each patient provides two Berlin grades since each hemisphere is assessed separately. The grade of the hemispheres might be correlated, although it is unknown if so and how strongly. Together with the ordinal scale of the outcome of interest, these issues motivate the development of new methods to deal with clustered data for ordinal variables. The boxplot in Figure 1 shows the data distributions. However, interpretation is barely possible since the visualization cannot include the potential correlation of the brain hemispheres per patient.

## 3 Statistical Model and Effects

**FIGURE 2:**
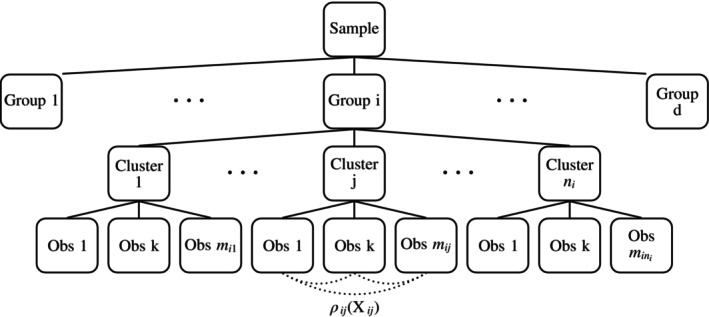
Structure of the clustered data in the several sample case.

A proper statistical model is needed for a reasonable framework for analyzing clustered data. We assume the design consists of d independent samples (groups). Independent random vectors can model the data (cluster) of sample i

(2) $$X_{ij}={\left(X_{ij1},\ldots ,X_{ijm_{ij}}\right)}^{⊺},i=1,\ldots ,d;j=1,\ldots ,n_{i}$$

with distributions $X_{ijk}\;∽\;F_{i}$, meaning that all individual observations within cluster j in group i follow the same marginal distribution $F_{i}$. The index j denotes the jth cluster in group i and $m_{ij}$ the number of observations within cluster j. Observations within the same cluster may be correlated, that is, the correlation matrix $\rho_{ij}(X_{ij})$ may be non‐diagonal. We do not assume any specific dependency structure within each cluster; the dependencies across the clusters may differ. The data structure is displayed in Figure 2. To account for metric, ordinal and even dichotomous data in a unified way, $F_{i}(x)$ denotes the normalized version of the distribution function, which is the average $F_{i}(x)=\frac{1}{2}\left(F_{i}^{-}(x)+F_{i}^{+}(x)\right)$ of the left‐continuous $\left(F_{i}^{-}=\mathbb{P}\left(X_{ijk}<x\right)\right)$ and right‐continuous $\left(F_{i}^{+}=\mathbb{P}\left(X_{ijk}\leq x\right)\right)$ versions of the distribution function of $X_{ijk}$ [25]. We furthermore denote by $n={\left(n_{1},\ldots ,n_{d}\right)}^{⊺}$ the vector containing the groupwise number of clusters and by $n=\sum_{i=1}^{d}n_{i}$, $M_{i}=\sum_{j=1}^{n_{i}}m_{ij}$ and $M=\sum_{i=1}^{d}\sum_{j=1}^{n_{i}}m_{ij}$ the total numbers of clusters, number of observations in group i and the total number of individual observations, respectively.

Model (2) does not contain any statistical parameters other than the distribution functions that could be used to define treatment effects. We therefore use them in the definition of a generalized relative effect

(3) $$\begin{array}{ll} p_{i} & =\int F_{\theta }dF_{i}=\mathbb{P}\left(G<X_{ijk}\right)+\frac{1}{2}\mathbb{P}\left(X_{ijk}=G\right),G\;∽\;F_{\theta }\\ & i=1,\ldots ,d \end{array}$$

of sample i with respect to a generalized mean distribution $F_{\theta }=\sum_{i=1}^{d}\theta_{i}F_{i}$, where $\theta ={\left(\theta_{1},\ldots ,\theta_{d}\right)}^{⊺}$ is a vector of non‐negative normalized weights $\theta_{i}$. The random variables $X_{ijk}$ and G are independent. Different choices of θ result in different effects and should be chosen cautiously. In clustered data designs, different weighing of the cluster sizes may be of interest, depending on the actual research question and whether the cluster sizes are informative or uninformative [26, 27, 28]. For instance, the effect $p_{i}$ generalizes the weighted relative effect $\left(\theta_{i}=\frac{M_{i}}{M}\right)$ [12]

$$\begin{array}{ll} p_{i,M} & =\int H_{M}dF_{i}=\mathbb{P}\left(Z<X_{ijk}\right)+\frac{1}{2}\mathbb{P}\left(X_{ijk}=Z\right)\\ Z & \;∽\;H_{N}=\frac{1}{M}\overset{d}{\underset{i=1}{\sum }}\overset{n_{i}}{\underset{k=1}{\sum }}\overset{m_{ik}}{\underset{s=1}{\sum }}F_{i},i=1,\ldots ,d \end{array}$$

and the unweighted relative effect $\left(\theta_{i}=\frac{1}{M}\right)$ [29]

$$\begin{array}{ll} \psi_{i} & =\int HdF_{i}=\mathbb{P}\left(Y<X_{ijk}\right)+\frac{1}{2}\mathbb{P}\left(X_{ijk}=Y\right)\\ Y & \;∽\;H=\frac{1}{d}\overset{d}{\underset{i=1}{\sum }}F_{i},i=1,\ldots ,d \end{array}$$

respectively. The weighted relative effect $p_{i,M}$ depends on sample sizes, and their allocations may end in surprising results [30]. If the cluster sizes are equal to one, the effect bases most of the joint rank procedures, including the Kruskal–Wallis test [31]. The unweighted relative effect $\psi_{i}$ does not depend on sample sizes; hence, it may be better suited to define a treatment effect in biomedical sciences. As recently discussed, $\psi_{i}$ does not come with advantages only; it changes when samples are combined (into one) [32]. The definition of $p_{i}$ in (3) allows flexible use of weights so that drawbacks from initial definitions may be reduced or even removed. Table 2 lists a selection of different weighing strategies.

**TABLE 2:** Different weighting strategies.

Weighting strategy	Description	Weight formula
1. Equal weighting (unweighted mean)	Each sample is given equal weight, regardless of the number of clusters or observations within each cluster.	$\theta_{i}=\frac{1}{d}$
2. Weighting by cluster size (weighted mean)	The total number of observations across all clusters in the sample weights each sample's contribution.	$\theta_{i}=\frac{\sum_{k=1}^{n_{i}}m_{ik}}{\sum_{i=1}^{d}\sum_{k=1}^{n_{i}}m_{ik}}$
3. Inverse cluster size weighting	Each sample is weighted inversely by the total number of observations across all clusters, giving more weight to samples with smaller total sizes.	$\theta_{i}=\frac{\frac{1}{\sum_{k=1}^{n_{i}}m_{ik}}}{\sum_{i=1}^{d}\frac{1}{\sum_{k=1}^{n_{i}}m_{ik}}}$
4. Variance‐based weighting	Each sample is weighted inversely by the overall variance across its clusters, giving more weight to samples with lower variance (in the case of metric data or using variance of the estimators).	$\theta_{i}=\frac{\frac{1}{\sum_{k=1}^{n_{i}}\sigma_{ik}^{2}}}{\sum_{i=1}^{d}\frac{1}{\sum_{k=1}^{n_{i}}\sigma_{ik}^{2}}}$
5. Hierarchical or multilevel weighting	A higher‐level criterion ($W_{i}$) determines each sample's weight, such as the number of clusters, overall variance, or other group‐level characteristics.	$\theta_{i}=\frac{W_{i}}{\sum_{i=1}^{d}W_{i}}$

Weighting schemes other than those mentioned above may be considered (e.g., data‐dependent or increasing/decreasing trends). Still, a detailed elaboration of such is far beyond the scope of this work and should be considered individually. Throughout the article, we will primarily use unweighted relative effects ($\theta_{i}=\frac{1}{d}$) because formulating null hypotheses in effects that depend on sample sizes (and their allocations) might result in issues and is debatable [30]. We do not use pairwise defined effects to not run into Efron's paradoxical dice leading to intransitive and paradoxical conclusions [19, 33, 34, 35]. The relative effects are interpreted as follows: if $p_{i}<\frac{1}{2}$, then the observations coming from $F_{i}$ tend to be smaller than those from $F_{\theta }$. If $p_{i}=p_{j}$, none of the observations tend to be smaller or larger. Therefore, no treatment effect between samples i and j refers to the situation $p_{i}=p_{j}$.

The definition of $p_{i}$ as in (3) covers a broad class of different effect descriptions. In the two‐sample case (d=2), the effects are similar to the ones discussed by Roy, Harrar, and Konietschke [16]. If $m_{ij}\equiv 1$, then the weighted and unweighted relative effects are identical to the underlying effects of the Kruskal–Wallis test and the pseudo‐rank procedures proposed by Brunner et al. [36]. The effects $p_{1},\ldots ,p_{d}$ are, however, unknown in practical applications and must be estimated from the data. Consistent estimators and their asymptotic distributions will be discussed in the next section.

### 3.1 Null Hypotheses

Research questions in several samples are manifold, often requiring specific hypotheses to be tested across different groups or conditions. Most of the existing rank procedures test the null hypothesis $H_{0}^{F}:\mathrm{CF}=0$ formulated in terms of the distribution functions $F={\left(F_{1},\ldots ,F_{d}\right)}^{\prime }$ [12]. However, this formulation of the null hypothesis is rather strict, and neither allows for variance heteroscedasticity nor the computation of confidence intervals for the relative effects. The alternative hypothesis is especially hard to interpret because the distributions are functions, making the alternatives functional. One powerful approach to overcome these limitations is through the formulation of null hypotheses as $H_{0}:\mathrm{Cp}=0$, where $p={\left(p_{1},\ldots ,p_{d}\right)}^{\prime }$ represents the vector of general relative effects, and C is an arbitrary contrast matrix. Each row of C represents a hypothesis about the relative effects. For example, the global null hypothesis

$$\begin{array}{l} H_{0}:p_{1}=\cdots =p_{d}\Leftrightarrow H_{0}:\mathrm{Cp}=0 \end{array}$$

can be equivalently formulated using the centering matrix $C=I_{d}-\frac{1}{d}1_{d}1_{d}^{\prime }$, where $I_{d}$ is the identity matrix of size d×d, and $1_{d}$ is a vector of ones of length d. This matrix centers the data by subtracting the mean. The research questions in general designs are manifold and, for example, many‐to‐one comparisons (comparing each group to a control) or all‐pairwise comparisons can be performed with:

$$\begin{array}{l} C=\left(\begin{array}{ccccc} 1 & -1 & 0 & \cdots & 0\\ 0 & 1 & -1 & \cdots & 0\\ \vdots & \vdots & \vdots & \ddots & \vdots \end{array}\right)\;\text{or}\;C=\left(\begin{array}{ccccc} 1 & -1 & 0 & \cdots & 0\\ 1 & 0 & -1 & \cdots & 0\\ \vdots & \vdots & \vdots & \ddots & \vdots \\ 0 & 1 & 0 & \cdots & -1 \end{array}\right) \end{array}$$

respectively. Under $H_{0}$, these specified contrasts are hypothesized to be zero, indicating no differences in the relative effects. Note that the assumed model in (2) and the null hypotheses formulated above are not equivalent to a linear model [4].

## 4 Point Estimators and Their Asymptotic Properties

In clustered data designs, the estimation of treatment effects (parameters) is more involved than in standard settings. Under the assumption of multivariate normality or if the data fit a generalized linear model, mixed models or generalized estimation equations are typically used for effect estimation. In principle, these estimators are weighted sums of the individual observations. Best linear unbiased and variance minimal estimators are available (e.g., least squares), which consider the correlation within the clusters. We follow these ideas in the following and provide a general, purely nonparametric framework for estimating $p_{i}$. Since $p_{i}$ is a function of the (cumulative) distribution functions, we begin by estimating them first and obtaining consistent estimators using the plug‐in method afterward.

### 4.1 Point Estimators

Consider the weighted version of the empirical distribution function ${\hat{F}}_{ij}^{\omega }(x)$ of the data in sample i,

(4) $$\begin{array}{ll} {\hat{F}}_{ijk}(x) & =1\left(X_{ijk}<x\right)+\frac{1}{2}1\left(X_{ijk}=x\right)\\ {\hat{F}}_{ij}^{\omega }(x) & =\overset{m_{ij}}{\underset{k=1}{\sum }}\omega_{ijk}{\hat{F}}_{ijk}(x)\\ {\hat{F}}_{i}^{\psi }(x) & =\overset{n_{i}}{\underset{j=1}{\sum }}\psi_{ij}{\hat{F}}_{ij}^{\omega }(x) \end{array}$$

where 1(·) denotes the indicator function. The weights $\omega_{ij}={\left(\omega_{ij1},\ldots ,\omega_{ijm_{ij}}\right)}^{⊺}$ and $\psi_{i}={\left(\psi_{i1},\ldots ,\psi_{in_{i}}\right)}^{⊺}$, are restricted to be non‐negative and normalized, that is, $\sum_{k=1}^{m_{ij}}\omega_{ijk}=1$ and $\sum_{j=1}^{n_{i}}\psi_{ij}=1$, respectively. They can be chosen arbitrarily as long as the requirements above are fulfilled. An estimator of $p_{i}$ in (3) is now readily available by replacing the unknown distribution functions with their empirical counterparts by

(5) $${\hat{p}}_{i}=\int {\hat{F}}_{\theta }d{\hat{F}}_{i}^{\psi }$$

where ${\hat{F}}_{\theta }(x)=\sum_{i=1}^{d}\theta_{i}{\hat{F}}_{i}^{\psi }(x)$ denotes the empirical counterpart of $F_{\theta }(x)$. It follows immediately from (5) that the estimator is a weighted sum of the placements ${\hat{F}}_{\theta }(X_{ijks})$, in particular, all data are taken into account. Choosing certain weights leads to well‐known cases, for example, choosing $\omega_{ijk}=m_{ij}^{-1}\forall k,\psi_{ij}=n_{i}^{-1}\;\forall \;j,\theta_{i}=d^{-1}\;\forall \;i$ results in an unweighted mean estimator

$${\hat{p}}_{i}^{\text{unweighted}}=\int {\hat{F}}_{\theta }d{\hat{F}}_{i}^{\psi }=\frac{1}{n_{i}}\overset{n_{i}}{\underset{j=1}{\sum }}\frac{1}{m_{ij}}\overset{m_{ij}}{\underset{k=1}{\sum }}{\hat{F}}_{\frac{1}{d}}(X_{ijk})$$

Note that ${\hat{F}}_{\frac{1}{d}}(X_{ijk})$ can be computed with pseudo‐ranks, whereas the weights $\omega_{ijk}=m_{ij}^{-1}\forall k,\psi_{ij}=\frac{m_{ij}}{M_{i}},\theta_{i}=\frac{M_{i}}{M}$ results in the weighted estimator

$${\hat{p}}_{i}^{\text{weighted}}=\int {\hat{F}}_{\theta }d{\hat{F}}_{i}^{\psi }=\frac{1}{M_{i}}\overset{n_{i}}{\underset{j=1}{\sum }}\overset{m_{ij}}{\underset{k=1}{\sum }}{\hat{F}}_{\theta }(X_{ijk})$$

which can be computed with the data's classical (mid‐) ranks, as Akritas and Brunner proposed [12]. Which weights to choose depends on the research question of interest and cannot be generally recommended. Next, the asymptotic (joint) distribution of the statistic $\sqrt{g(n)}\left(\hat{p}-p\right)$ will be established, in which the factor g(n) ensures that the limiting distribution is non‐degenerate. In the presented weighting schemes, the factor g(n) is either g(n)=n (unweighted estimator) or g(n)=M (weighted estimator). The vectors are defined as $p={\left(p_{1},\ldots ,p_{d}\right)}^{⊺}$ and $\hat{p}={\left({\hat{p}}_{1},\ldots ,{\hat{p}}_{d}\right)}^{⊺}$.

### 4.2 Asymptotic Results

Furthermore, to introduce the theoretical results, we consider the following mild assumptions:

A1. $m_{ij}<\infty \forall \;i,j$;
A2. $n\;\to \;\infty \Rightarrow n_{i}\;\to \;\infty \;\forall \;i$;
A3. $n\to \infty \Rightarrow \frac{n_{i}}{n}\leq c\in \mathbb{R}\;\forall \;i$;
A4. n→∞⇒g(n)→∞;
A5. $n\to \infty \Rightarrow g(n)\cdot \psi_{ij}\leq c\in \mathbb{R}\;\forall \;i,j$;
A6. $n\to \infty \Rightarrow \psi_{ij}\to 0\;\forall \;i,j$.

The first Assumption A1 states that the number of individual observations per cluster is uniformly bounded and finite. The next two assumptions (A2 and A3) assume that if the sample size n grows considerably more extensive, the group‐wise sample sizes grow in the same way. Assumption A4 applies restrictions to our inflation function. However, in the unweighted case, this assumption is satisfied by definition, and in the weighted case, it follows immediately from the former assumptions. Finally, the last two assumptions restrict our weights. Assumption A5 states that the product of the inflating function and the weight is less than some real constant, as n→∞. In the unweighted case, this is follows from A2. For the weighted case, this follows from Assumption A1. The last assumption states that the weights converge to 0 as n→∞, which follows immediately from A1 in the weighted and A2 in the unweighted case. Thus, in our two exemplary weighting schemes, the Assumptions A4–A6 follow from the former three assumptions. In turn, statistical practice makes the first three assumptions mild and reasonable. To this end, it will be shown in Theorem 1that $\sqrt{g(n)}\left(\hat{p}-p\right)$ has asymptotically (n→∞) the same distribution as

(6) $$A_{g(n)}=\sqrt{g(n)}\overset{d}{\underset{i=1}{\sum }}\overset{n_{i}}{\underset{j=1}{\sum }}\psi_{ij}A_{ij}$$

where

Aij=(A1ij,…,Adij)⊺Ahij=∑s=1s≠idθsYij(s)h=i,−θiYij(h)h≠i,andYab(c)=∑k=1mabωabkFc(Xabk)

Theorem 1 (Asymptotic equivalence) *If* n→∞ *such that A1–A6 hold, then*

$${\left|\left|\sqrt{g(n)}\left(\hat{p}-p\right)-\sqrt{g(n)}\left(1-2p+\overset{d}{\underset{i=1}{\sum }}\overset{n_{i}}{\underset{j=1}{\sum }}\psi_{ij}A_{ij}\right)\right|\right|}_{2}^{2}\;\to \;0$$

Appendix A.2 in Supporting Information.

It follows from Theorem 1 that $\sqrt{g(n)}\left(\hat{p}-p\right)$ has, asymptotically, the same distribution as the random vector $A_{g(n)}$ defined in Equation (6). Its asymptotic normality will be established in the next theorem.

Theorem 2 *Under Assumptions A1–A6, the rank statistic* $\sqrt{g(n)}\left(\hat{p}-p\right)$ *follows a multivariate normal distribution with expectation* 0 *and covariance matrix* $\sum =𝕍\text{ar}\left(A_{g(n)}\right)$.

Note that $A_{g(n)}$ is a sum of independent random vectors, and therefore, its (asymptotic) multivariate normality follows from the Cramér–Wold technique. The Lindeberg condition is fulfilled because the components of the vectors are uniformly bounded.

However, the covariance matrix ∑ is unknown and must be estimated from the data. A consistent estimator will be introduced in the following subsection.

### 4.3 Estimation of the Covariance Matrix

By independence of the random vectors $A_{ij}$ and $A_{ij^{\prime }},j\neq j^{\prime }$, it follows that

(7) $$\begin{array}{ll} 𝕍\text{ar}\left(\sqrt{g(n)}\left(\hat{p}-p\right)\right) & ≑𝕍\text{ar}\left(\sqrt{g(n)}\overset{d}{\underset{i=1}{\sum }}\overset{n_{i}}{\underset{j=1}{\sum }}\psi_{ij}A_{ij}\right)\\ & =g(n)\overset{d}{\underset{i=1}{\sum }}\overset{n_{i}}{\underset{j=1}{\sum }}\psi_{ij}^{2}\sum_{ij}\\ & \text{where}\;\sum_{ij}=𝕍\text{ar}\left(A_{ij}\right) \end{array}$$

We proceed with defining an estimator of $\sum_{ij}$ by

$${\hat{\sum }}_{ij}^{*}=\left(A_{ij}-{\bar{A}}_{i•}^{*}\right){\left(A_{ij}-{\bar{A}}_{i•}^{*}\right)}^{⊺},{\bar{A}}_{i•}^{*}=\overset{n_{i}}{\underset{j=1}{\sum }}\psi_{ij}A_{ij}$$

However, replacing $\sum_{ij}$ with ${\hat{\sum }}_{ij}^{*}$ in (7) results in a biased estimator of ∑. Note that the bias occurs from the weighted linear combination of ${\hat{\sum }}_{ij}^{*}$. Using the following modifications

$$\begin{array}{ll} \kappa_{ij} & =1-2\psi_{ij}+\overset{n_{i}}{\underset{j^{\prime }=1}{\sum }}\psi_{ij^{\prime }}^{2}\\ {\hat{\sum }}^{*} & =g(n)\overset{d}{\underset{i=1}{\sum }}\overset{n_{i}}{\underset{j=1}{\sum }}\psi_{ij}^{2}\kappa_{ij}^{-1}{\hat{\sum }}_{ij}^{*} \end{array}$$

results in an unbiased estimator of ∑. The proof can be found in Appendix A.3 in Supporting Information. However, the estimator ${\hat{\sum }}^{*}$ is not observable, since the random variables $Y_{ab}^{\left(c\right)}$ depend on the unknown distribution function $F_{c}$. We therefore replace them with their empirical (observable) counterparts ${\hat{Y}}_{ab}^{\left(c\right)}=\sum_{k=1}^{m_{ab}}\omega_{abk}{\hat{F}}_{c}^{\psi }\left(X_{abk}\right)$ throughout. Let

A^ij=(A^1ij,…,A^dij)⊺,A^hij=∑s=1s≠idθsY^ij(s)h=i,−θiY^ij(h)h≠i,andY^ab(c)=∑k=1mabωabkF^c(Xabk)

denote the observable empirical counterparts of the random vectors $A_{ij}$ and let

$${\hat{\sum }}_{ij}=\left({\hat{A}}_{ij}-{\bar{\hat{A}}}_{i•}\right){\left({\hat{A}}_{ij}-{\bar{\hat{A}}}_{i•}\right)}^{⊺},{\bar{\hat{A}}}_{i•}=\overset{n_{i}}{\underset{j=1}{\sum }}\psi_{ij}{\hat{A}}_{ij}$$

Finally, it will be shown in the next theorem that

(8) $$\hat{\sum }=g(n)\overset{d}{\underset{i=1}{\sum }}\overset{n_{i}}{\underset{j=1}{\sum }}\psi_{ij}^{2}\kappa_{ij}^{-1}{\hat{\sum }}_{ij}$$

is a consistent estimator of ∑.

Theorem 3 (Consistency of the variance estimator) *Let* $\hat{\sum }$ *as given in (* *8* *). Then, if Assumptions A1–A6 hold,*

$${\left|\left|\hat{\sum }-\sum \right|\right|}_{2}^{2}\to 0$$

Appendix A.4 in Supporting Information.

Since we have derived the limiting distribution and asymptotic parametrization of our point estimator $\hat{p}$, we proceed with hypothesis testing and confidence intervals in the following section.

## 5 Hypothesis Tests

The following section will establish test procedures for testing the null hypothesis $H_{0}:\mathrm{Cp}=0$. Here, C denotes a (general) contrast matrix of dimension dim(C)=q×d, where q refers to the number of individual contrasts $c_{l}^{⊺}p$. The procedures are not restricted to a specific type of contrast as long as the contrast matrix satisfies basic properties: full row‐rank and rows must sum up to zero ($c_{l}^{⊺}1=0$). In the following, we propose global test procedures (quadratic forms) and multiple contrast test procedures (MCTPs). While global test procedures can be used for testing global null hypotheses only, the MCTP can be used for testing individual and local null hypotheses as well as for the computation of simultaneous confidence intervals (SCI). From a practical point of view, the MCTPs provide more information than quadratic forms and are preferably applied in statistical practice. Strictly speaking, the two methods are not comparable because the global tests are quadratic while the MCTPs are linear. However, both methods can be used to test the same (global) null hypothesis, and we study them side by side. The respective subsections will discuss further differences between the methods and their pros and cons.

### 5.1 Wald‐Type Test

The first statistic that we present is the Wald‐type test statistic

(9) $$Q^{\left(w\right)}=g(n){\hat{p}}^{⊺}C^{⊺}{\left(C\hat{\sum }C^{⊺}\right)}^{+}C\hat{p}$$

where ${\left(\cdot \right)}^{+}$ denotes a generalized inverse (e.g., Moore–Penrose inverse) of a matrix. Under $H_{0}:\mathrm{Cp}=0$, we approximate the distribution of $Q^{\left(w\right)}$ by a $\chi^{2}$ distribution with $\text{rk}\left(\hat{M}\right)$ degrees of freedom, where $\hat{M}=C\hat{\sum }C^{⊺}$. Note that the approximation requires the further regularity condition $\text{rk}\left(\hat{M}\right)\overset{\mathbb{P}}{\to }\text{rk}\left(M\right)$, because $\hat{\sum }$ may be singular and if C does not have a full column rank, ${\hat{M}}^{+}$ does in general not converge in probability to the matrix $M^{+}={\left(C\sum C^{⊺}\right)}^{+}$ [36, 37]. The test is known to be quite liberal unless sample sizes are (very) large [36, 37, 38, 39]. The liberality arises in small samples because the limiting distribution does not account for the randomness of the estimator $\hat{\sum }$. Furthermore, estimating the generalized inverse matrix is a problem of its own. However, we will present simulation results for this test in Section 7 for completeness.

### 5.2 ANOVA‐Type Test

Due to the liberal behavior of the Wald‐type statistic in small sample sizes, we propose the ANOVA‐type statistic

(10) $$Q^{\left(a\right)}=\frac{g(n)}{\text{tr}\left(M\hat{\sum }\right)}{\hat{p}}^{⊺}M\hat{p}$$

as a competitor. Here, $M=C^{⊺}{\left({\mathrm{CC}}^{⊺}\right)}^{-}C$ denotes the projection matrix and tr(·) the trace of a square matrix, respectively. In clustered data designs, approximating the null distribution of $Q^{\left(a\right)}$ is pretty involved. Rubarth et al. [29] propose to approximate its distribution by $\chi_{{\hat{f}}_{1}}^{2}/{\hat{f}}_{1}$ distribution with

(11) $${\hat{f}}_{1}=\frac{{\left(\text{tr}\left(M\hat{\sum }\right)\right)}^{2}}{\text{tr}\left(M\hat{\sum }M\hat{\sum }\right)}$$

degrees of freedom. Note that the sampling distribution of the denominator $\text{tr}\left(M\hat{\sum }\right)$ is not accounted for within the approximation scheme, resulting in inflated type‐I error rates in small samples. We therefore propose to follow the ideas by Brunner et al. [36] in univariate models and approximate the distribution of $Q^{\left(a\right)}$ by a central $F_{{\hat{f}}_{1},{\hat{f}}_{2}}$ distribution [36]. For the computation of ${\hat{f}}_{2}$, let $R_{ijk}=n{\hat{F}}_{\theta }(X_{ijk})+\frac{1}{2}$ denote the global rank of an individual observation and let $R_{ijk}^{\left(i\right)}=M_{i}{\hat{F}}_{i}^{\psi }(X_{ijk})+\frac{1}{2}$ denote the group‐wise internal rank of $X_{ijk}$ among all $M_{i}$ observations in sample i. Furthermore, define the mean ranks as ${\bar{R}}_{i\cdot }=\sum_{j=1}^{n_{i}}\psi_{ij}\sum_{k=1}^{m_{ij}}\omega_{ijk}R_{ijk}$. Finally, let

(12) $$\begin{array}{ll} S_{i}^{2} & =\frac{1}{n_{i}-1}\overset{n_{i}}{\underset{j=1}{\sum }}{\left(\overset{m_{ij}}{\underset{k=1}{\sum }}\omega_{ijk}\left[R_{ijk}-R_{ijk}^{\left(i\right)}\right]-{\bar{R}}_{i\cdot }+\frac{M_{i}+1}{2}\right)}^{2}\\ {\hat{f}}_{2} & =\frac{{\left(\sum_{i=1}^{d}\frac{S_{i}^{2}}{n-n_{i}}\right)}^{2}}{\sum_{i=1}^{d}{\left(\frac{S_{i}^{2}}{n-n_{i}}\right)}^{2}{\left(n_{i}-1\right)}^{-1}} \end{array}$$

The chosen degree of freedom coincides with one proposed by Brunner et al. [36] if $m_{ij}=1\forall i,j$, that is, if literally, no clusters are present. Additionally, in the case of two samples (d=2) and $m_{ij}=1$, the test coincides with the Brunner–Munzel test [40]. Note that ${\hat{f}}_{2}\to \infty$ as n→∞ and therefore, the approximation is asymptotically correct. The Wald‐type and ANOVA‐type tests can only be used to test global null hypotheses. Testing individual null hypotheses and making local inferences is not possible. We, therefore, introduce and discuss multiple contrast test procedures in the next section.

### 5.3 Multiple Contrast Test Procedures (Mctps)

We proposed two global test procedures for testing $H_{0}:\mathrm{Cp}=0$ in the former subsections. If $H_{0}$ gets rejected by any of these procedures, then *any* of the d treatments (conditions) differ at significance level α. The tests cannot provide information on which of them differ. This information is, however, key and of primary importance for the researchers. Instead, testing multiple null hypotheses and estimating simultaneous confidence intervals for the treatment effects of interest are fundamental in modern data evaluations, especially in early phases, for example, pre‐clinical or clinical trials [38, 41, 42]. We therefore propose multiple contrast test procedures for testing $H_{0}$ with the family of null hypotheses

(13) $$\Omega =\left\{l\in [1,\ldots ,q],q\in \mathbb{N}:H_{0}^{\left(l\right)}:c_{l}^{⊺}p=0\right\}$$

where $c_{l}^{⊺}$ denotes the lth row vector of the contrast matrix C. In order to test $H_{0}^{\left(l\right)}:c_{l}^{⊺}p=0$, consider the test statistic

$$T_{l}=\sqrt{\frac{g(n)}{{\hat{\Upsilon }}_{ll}}}c_{l}^{⊺}\left(\hat{p}-p\right)$$

where ${\hat{\Upsilon }}_{ll}$ denotes the lth diagonal element of $\hat{ϒ}=C\hat{\sum }C^{⊺}$. Note that the test statistics $T_{l}$ and $T_{l^{\prime }}$ are not necessarily independent and ignoring their dependency typically results in loss of efficiency within the data analysis. We therefore collect them in the vector $T={\left(T_{1},\ldots ,T_{q}\right)}^{⊺}$, which follows, asymptotically, as n→∞, a multivariate normal distribution with expectation 0 and correlation matrix

$$R=\text{diag}{\left(ϒ\right)}^{-1/2}ϒ\text{diag}{\left(ϒ\right)}^{-1/2}$$

For large sample sizes, the individual null hypothesis $H_{0}^{\left(l\right)}:c_{l}^{⊺}p=0$ will be rejected at multiple level α (family wise error rate), if

(14) $$\left|T_{l}|\geq z_{1-\alpha }(R\right)$$

where $z_{1-\alpha }(R)$ denotes the two‐sided (1−α)∗100% equi‐coordinate quantile from the N(0,R) distribution. A compatible simultaneous confidence interval for the treatment of interest $\delta_{l}=c_{l}^{⊺}p$ is given by

(15) $$CI_{l}=\left[c_{l}^{⊺}\hat{p}\mp \frac{z_{1-\alpha }(R)}{\sqrt{g(n)}}\sqrt{{\hat{\Upsilon }}_{ll}}\right]$$

Finally, for large sample sizes, the global null hypothesis will be rejected, if

(16) $$Q^{\left(m\right)}=\max\{|T_{1}|,\ldots ,|T_{q}|\}\geq z_{1-\alpha }(R)$$

The correlation matrix R is, however, unknown and must be estimated in data evaluations. We recommend to replace R with the consistent estimator

(17) $$\hat{R}=\text{diag}{\left(\hat{ϒ}\right)}^{-1/2}\hat{ϒ}\text{diag}{\left(\hat{ϒ}\right)}^{-1/2}$$

in ((14), (15), (16)), respectively. Extensive simulation studies indicate a liberal behavior of the normal approximation when sample sizes are smaller than 30 (depending on the number of groups and the chosen contrast). For small sample sizes, we approximate the distribution of T by a central multivariate t‐distribution with ν degrees of freedom and correlation matrix $\hat{R}$ [41, 43]. Following the ideas of Gao et al. [43] and Konietschke et al. [41], let $A_{ij}$ be as in Theorem 1and let ${\hat{\Lambda }}_{lij}=c_{l}^{⊺}{\hat{A}}_{ij}$. Furthermore, let

$$\begin{array}{ll} {\bar{\Lambda }}_{li\cdot } & =\overset{n_{i}}{\underset{j=1}{\sum }}\psi_{ij}{\hat{\Lambda }}_{lij},{\hat{v}}_{li}^{2}=\overset{n_{i}}{\underset{j=1}{\sum }}\psi_{ij}\kappa_{ij}^{-1}{\left({\hat{\Lambda }}_{lij}-{\bar{\Lambda }}_{li\cdot }\right)}^{2},\text{and}\\ {\hat{\nu }}_{l} & =\frac{{\left(\sum_{i=1}^{d}{\hat{v}}_{li}^{2}\right)}^{2}}{\sum_{i=1}^{d}\frac{1}{n_{i}-1}{\hat{v}}_{li}^{4}} \end{array}$$

denote a Satterthwaite–Welch‐type degree of freedom for each contrast. We chose the degree of freedom of the MCTP statistic conservatively as the smallest value larger than one, that is,

(18) $$\hat{\nu }=\max\left\{1,\underset{l\in [1,\ldots ,q]}{\min}{\hat{\nu }}_{l}\right\}$$

For small sample sizes, we replace the critical values $z_{1-\alpha }(\hat{R})$ (and p values) with the equi‐coordinate quantile $t_{1-\alpha }(\hat{R})$ from a multivariate t‐distribution with $\hat{\nu }$ degrees of freedom and correlation matrix $\hat{R}$ in ((14), (15), (16)), respectively. Since the family of null hypotheses Ω and test statistics T constitute a joint testing family, the simultaneous test procedures control the family‐wise error rate in the strong sense [44].

#### 5.3.1 Range Preserving Confidence Intervals

The SCIs discussed above are not necessarily range‐preserving, that is, the lower/upper limits may be smaller/larger than [−1,1], respectively. To overcome the drawback, we follow Konietschke et al. [41] and use the Fisher‐transformation function to compute range‐preserving SCIs. Throughout the following, we assume that the contrasts are normed, that is, $\left|c_{li}\right|\leq 1\forall i,l$. It follows from the multivariate delta method,

$$\sqrt{g(n)}(\zeta (\hat{\delta })-\zeta (\delta ))\overset{d}{\to }N(0,\Gamma )$$

where

$$\begin{array}{ll} \Gamma & =\eta ϒ\eta^{⊺},\;\eta ={\left(\text{diag}\left({\left(1-\delta_{l}^{2}\right)}^{-1}\right)\right)}_{l\in \{1,\ldots ,q\}}\\ \delta & ={\left(\delta_{l}\right)}_{l\in \{1,\ldots ,q\}},\;\hat{\delta }={\left({\hat{\delta }}_{l}\right)}_{l\in \{1,\ldots ,q\}}\\ \delta_{l} & =c_{l}^{⊺}p,\;{\hat{\delta }}_{l}=c_{l}^{⊺}\hat{p}\\ \zeta (x_{i}) & =\frac{1}{2}\log\left(\frac{1+x_{i}}{1-x_{i}}\right),\;\zeta (x)={\left(\zeta (x_{i})\right)}_{i\in \dim\left(x\right)} \end{array}$$

Here, η is the Jacobian matrix of ζ(δ). The unknown parameters Γ and η can be estimated with their empirical counterparts $\hat{\Gamma }$, $\hat{\eta }={\left(\text{diag}\left({\left(1-{\hat{\delta }}_{i}^{2}\right)}^{-1}\right)\right)}_{i\in \{1,\ldots ,q\}}$. For testing the null hypothesis $H_{0}:\mathrm{Cp}=0$, consider the maximum statistic ${\tilde{Q}}^{\left(m\right)}=\max_{l}\left|\tilde{T}\right|$, where $\tilde{T}={\left({\tilde{T}}_{1},\ldots ,{\tilde{T}}_{q}\right)}^{\prime }$ denotes the vector of Fisher‐transformed test statistics

(19) $${\tilde{T}}_{l}=\frac{\sqrt{g(n)}\left(\zeta ({\hat{\delta }}_{l})-\zeta (\delta_{l})\right)}{\sqrt{{\hat{\Gamma }}_{ll}}}$$

The vector of test statistics $\tilde{T}$ follows, asymptotically, a multivariate normal distribution with expectation 0 and correlation matrix R. Note that the correlation of the test statistics is not affected by the transformation function and therefore R remains the same. We now can construct SCIs for the transformed effects, where $c^{\left(m\right)}$ are critical values of the multivariate normal‐ or t‐distribution,

$$\begin{array}{ll} \tau_{\text{low}}^{\left(l\right)} & =\zeta ({\hat{\delta }}_{l})-c^{\left(m\right)}(\alpha )\frac{1}{\sqrt{g(n)}}\sqrt{{\hat{\Gamma }}_{ll}}\\ \tau_{\text{up}}^{\left(l\right)} & =\zeta ({\hat{\delta }}_{l})+c^{\left(m\right)}(\alpha )\frac{1}{\sqrt{g(n)}}\sqrt{{\hat{\Gamma }}_{ll}} \end{array}$$

and find that the SCIs

$${\tilde{CI}}_{l,a}^{\left(c\right)}=\left[\zeta^{-1}(\tau_{\text{low}}^{\left(l\right)}),\zeta^{-1}(\tau_{\text{up}}^{\left(l\right)})\right]$$

are range‐preserving, because the inverse of the Fisher‐transformation $\zeta^{-1}(y)=\frac{\exp(2y)-1}{\exp(2y)+1}\in (0,1)$, by construction.

## 6 Special Cases

### 6.1 Testing Null Hypothesis of Equal Distribution Functions

Throughout, we have discussed nonparametric inference methods for relative effects. Rank procedures for testing equality of distribution functions by $H_{0}^{F}:\mathrm{CF}=0$ are well established in statistical sciences [12]. In some situations, for example, in sample size planning, they might even be of primary interest. We therefore develop statistical methods for testing $H_{0}^{F}$ as well. It follows from Theorem 1, that

$$\begin{array}{ll} & \sqrt{g(n)}C(\hat{p}-p)\\ & ≑\sqrt{g(n)}\left(C\int F_{\theta }d{\hat{F}}^{\psi }-C\int Fd{\hat{F}}_{\theta }+C\left(1-2p+A\right)\right) \end{array}$$

and thus, under $H_{0}^{F}:\mathrm{CF}=0$,

$$\begin{array}{ll} \sqrt{g(n)}C\hat{p} & ≑\sqrt{g(n)}C\int F_{\theta }d{\hat{F}}^{\psi }=\sqrt{g(n)}C{\bar{Y}}_{•}\\ & \text{where}{\bar{Y}}_{•}={\left({\bar{Y}}_{1\cdot },\ldots ,{\bar{Y}}_{d\cdot }\right)}^{⊺} \end{array}$$

is a vector of the sum of independent random variables

$${\bar{Y}}_{i\cdot }=\overset{n_{i}}{\underset{j=1}{\sum }}\psi_{ij}Y_{ij}\text{and}Y_{ij}=\overset{m_{ij}}{\underset{k=1}{\sum }}\omega_{ijk}F_{i}(X_{ijk})$$

respectively. Therefore, $\sqrt{g(n)}C\hat{p}$ follows, asymptotically, if $n\to \infty :\frac{n}{n_{i}}\leq N_{0}<\infty$ and $m_{ijk}<K_{0}<\infty$, a multivariate normal distribution with expectation 0 and covariance matrix $V={C\sum }_{F}C^{⊺}$ under $H_{0}^{F}$, where

$$\sum_{F}=\text{diag}\left(\sigma_{1}^{2},\ldots ,\sigma_{d}^{2}\right),\sigma_{i}^{2}=\mathrm{Var}({\bar{Y}}_{i\cdot })$$

Thus, the computation of the covariance matrix of $\sqrt{g(n)}C\hat{p}$ simplifies tremendously under $H_{0}^{F}$. If the random variables $Y_{ij}$ were observable, then an unbiased estimator of $\sigma_{i}^{2}$ would be given by

$${\tilde{\sigma }}_{i}^{2}=\overset{n_{i}}{\underset{j=1}{\sum }}\psi_{ij}^{2}\kappa_{ij}^{-1}{\left(Y_{ij}-{\bar{Y}}_{i\cdot }^{*}\right)}^{2},\kappa_{ij}=1-2\psi_{ij}+\overset{n_{i}}{\underset{j^{\prime }=1}{\sum }}\psi_{ij^{\prime }}^{2}$$

(Proof is given in Appendix A.5 in Supporting Information). The random variables are, however, not observable, and therefore, ${\tilde{\sigma }}_{i}^{2}$ cannot be computed in practice. Therefore, we replace the unknown variables $Y_{ij}$ with their empirical counterparts ${\hat{Y}}_{ij}=\sum_{k=1}^{m_{ij}}\omega_{ijk}{\hat{F}}_{i}(X_{ijk})$ in the computations above. Test procedures for testing $H_{0}^{F}:\mathrm{CF}=0$ can now be derived analogously.

### 6.2 One Observation Per Cluster

If we induce the restriction that only one observation per cluster is apparent ($m_{ij}=1\forall i,j$), this is effectively the same as no clusters at all. The new statistical model reduces to

(20) $$X_{ij}\;∽\;F_{i},i=1,\ldots ,d;j=1,\ldots ,n_{i}$$

Therefore, the estimators also reduce, such that ${\hat{F}}_{ij}^{\omega }$ can be abandoned, since $\omega_{ijk}$ must be 1 and $m_{ij}=1$. The following estimators remain:

(21) $$\begin{array}{ll} {\hat{F}}_{ij}(x) & =1\left(X_{ij}<x\right)+\frac{1}{2}1\left(X_{ij}=x\right)\\ {\hat{F}}_{i}^{\psi }(x) & =\overset{n_{i}}{\underset{j=1}{\sum }}\psi_{ij}{\hat{F}}_{ij}(x) \end{array}$$

The reference distribution function and its estimator are identical to the cases with $m_{ij}>1$. However, note that the most suitable choice for $\psi_{ij}$ would be $\psi_{ij}=n_{i}^{-1}$, leading to the standard definition of the empirical cumulative distribution function. Certain weighting schemes have been discussed thoroughly in the literature. Choosing $\theta_{i}=\frac{1}{d}\forall i$ leads to the pseudo‐rank procedures discussed by Brunner and Puri [45], Gao et al. [43], Konietschke, Hothorn, and Brunner [41] and Brunner et al. [36]. On the other hand, choosing $\theta_{i}=\frac{n_{i}}{n}$ leads to the standard rank procedures, as discussed by Akritas, Arnold, and Brunner [31].

### 6.3 Two‐Sample Case

The two‐sample case has been elaborated thoroughly by Roy, Harrar, and Konietschke [16]. We note that their procedure differs slightly from ours although similar. As they have only d=2, they choose the pairwise relative effect $p=\int F_{1}dF_{2}$. This is not identical to our approach since we have in this special case two relative effects $p_{1}=\theta_{1}\frac{1}{2}+\theta_{2}\int F_{2}dF_{1}$ and $p_{2}=\theta_{1}\int F_{1}dF_{2}+\theta_{2}\frac{1}{2}$. However, the similarity between p and $p_{2}$ is obvious.

If we introduce the additional restriction of $m_{ij}=1$ as in the former subsection, the test reduces to the Brunner–Munzel test. In turn, the Brunner–Munzel test reduces to the Mann–Whitney test, if we restrict the distribution functions to be identical, that is, $F_{1}=F_{2}$ [40, 46].

## 7 Simulation Results

Although we provide finite sample size corrections for the developed methods, our results still rely on asymptotic theory. In many applications and especially in pre‐clinical trials, however, sample sizes are (rather) small and limited. To investigate whether the Wald‐type test in (9), the ANOVA‐type test in (10), and the Fisher‐transformed MCTPs in (19) are applicable in these situations and control the nominal type‐I error rate (and have an acceptable power to detect selected alternatives), we conduct extensive simulation studies in various models. For each scenario, we used $10^{4}$ simulations runs, that is, $10^{4}$ independently generated data sets. Data have been generated from

$$X_{ij}={\left(X_{ijk},\ldots ,X_{ijm_{ij}}\right)}^{⊺}∼F_{i},i=1,\ldots ,d;j=1,\ldots ,n_{i}$$

Here, $F_{i}(x)$ denotes a $m_{ij}$‐variate multivariate distribution function with identical marginals. We select a few realistic scenarios from many possible designs to provide relevant information for practitioners. These include balanced and unbalanced designs, independent and dependent replicates, different correlation structures and strengths, different distributions, as well as different cluster‐ and sample sizes. Table 3 provides an overview of the simulation settings selected for this article. All models were run for cluster sizes $m_{ij}\in {ℳ}_{ij}=\{2,\ldots ,15\}$, sample sizes $n_{i}\in {𝒩}_{i}=\{12,15,20\}$ and number of groups d∈𝒟={2,3,4,5}. In unbalanced designs, we differ between mild and severe imbalances. The former is constructed in the following way: for the kth element of ${ℳ}_{ij}$, each $m_{ij}$ is drawn randomly from the vector $\left(\max\{{ℳ}_{ij}(k)-3,2\},\ldots ,{ℳ}_{ij}(k)+3\right)$. On the other hand, the severe imbalance is constructed such that there are $n_{i}-2$ clusters with cluster size ${ℳ}_{ij}(k)$, one cluster with size $m_{ij}=2$ and one with $m_{ij}=15$. We follow a similar principle when constructing the sample sizes in imbalanced designs; mild imbalance is generated by drawing $n_{i}$ randomly from the vector $\left({𝒩}_{i}(k^{\prime })-3,\ldots ,{𝒩}_{i}(k^{\prime })+3\right)$ and for the severe imbalance, we have one group with sample size $n_{i}=8$, one with $n_{i}=25$ and remaining groups have sample size ${𝒩}_{i}(k^{\prime })$, where $k^{\prime }$ is the $k^{\prime }$th element of ${𝒩}_{i}$. Considering the intracluster dependency, we consider homogeneous (Ho) and heterogeneous (He) correlation structures. The former is generated by an equi‐correlation matrix for each cluster, such that—within a cluster—the correlation between elements is always the same, which is $\rho_{\max}$ in our simulation study. On the other hand, in a heterogeneous setting, we compute the outer product $\rho_{ij}={\left(\sqrt{\rho_{r}^{\left(u\right)}}\right)}_{r\in [1,\ldots ,m_{ij}]}{\left(\sqrt{\rho_{r}^{\left(u\right)}}\right)}_{r\in [1,\ldots ,m_{ij}]}^{⊺}$, where $\rho_{r}^{\left(u\right)}\;∽\;U(\rho_{\min},\rho_{\max})$. Afterward, we set the diagonal elements of $\rho_{ij}$ to 1.

**TABLE 3:** Models for Type‐I error simulations.

	Marginal distribution	Imbalance	Variances	Correlation	Weighting scheme
Model 1.1	N(0,1)	None	Identical	None	Pseudo‐Rank
Model 1.2	N(0,1)	None	Identical	Ho, $\rho_{\max}=0.85$	Pseudo‐Rank
Model 1.3	N(0,1)	None	Identical	He, $\rho_{\min}=0.05$, $\rho_{\max}=0.35$	Pseudo‐Rank
Model 2	Beta(2,5)	None	Identical	Ho, $\rho_{\max}=0.35$	Pseudo‐Rank
Model 3	Pois(5)	None	Identical	He, $\rho_{\min}=0.6$, $\rho_{\max}=0.85$	Pseudo‐Rank
Model 4.1	N(0,1)	Mild	Identical	He, $\rho_{\min}=0.05$, $\rho_{\max}=0.35$	Pseudo‐Rank
Model 4.2	N(0,1)	Mild	Identical	He, $\rho_{\min}=0.05$, $\rho_{\max}=0.35$	Rank
Model 5	Bin(5,0.6)	Mild	Identical	Ho, $\rho_{\max}=0.85$	Pseudo‐Rank
Model 6.1	N(0,1)	Severe	Identical	He, $\rho_{\min}=0.6$, $\rho_{\max}=0.85$	Pseudo‐Rank
Model 6.2	N(0,1)	Severe	Identical	He, $\rho_{\min}=0.6$, $\rho_{\max}=0.85$	Rank
Model 7	Pois(5)	Severe	Identical	He, $\rho_{\min}=0.05$, $\rho_{\max}=0.35$	Pseudo‐Rank
Model 8.1	$N(0,\sigma_{i})$	None	$\sigma_{i}=0.1\cdot i^{2}$	He, $\rho_{\min}=0.6$, $\rho_{\max}=0.85$	Pseudo‐Rank
Model 8.2.1	$N(0,\sigma_{i})$	Mild	$\sigma_{i}=0.1\cdot i^{2}$	He, $\rho_{\min}=0.6$, $\rho_{\max}=0.85$	Pseudo‐Rank
Model 8.2.2	$N(0,\sigma_{i})$	Mild	$\sigma_{i}=0.1\cdot i^{2}$	He, $\rho_{\min}=0.6$, $\rho_{\max}=0.85$	Rank
Model 8.3.1	$N(0,\sigma_{i})$	Severe	$\sigma_{i}=0.1\cdot i^{2}$	He, $\rho_{\min}=0.6$, $\rho_{\max}=0.85$	Pseudo‐Rank
Model 8.3.2	$N(0,\sigma_{i})$	Severe	$\sigma_{i}=0.1\cdot i^{2}$	He, $\rho_{\min}=0.6$, $\rho_{\max}=0.85$	Rank

To simulate the power of the tests, we again selected a few scenarios deemed realistic (see Table 4). The number of groups (𝒟) and the sample sizes (${𝒩}_{i}$) are identical to the scenarios of the type‐I error simulations. However, we fixed the cluster sizes to $m_{ij}^{*}=4$. Note that in imbalanced designs, the actual cluster size $m_{ij}$ varies from 2 to 7 due to how we construct imbalance. Finally, we use a shift factor r∈{0.05,0.1,…,0.5} to generate data under the alternative hypothesis $H_{1}$.

**TABLE 4:** Models for Type‐II error simulations.

	Marginal distribution	Parameters	Imbalance	Correlation	Weighting scheme
Model 1.1	Normal	${\left\{\mu_{i}=r\cdot i,\sigma_{i}=0.7+0.1\cdot i\right\}}_{i\in [1,d]}$	Mild	None	Pseudo‐Rank
Model 1.2	Normal	${\left\{\mu_{i}=r\cdot i,\sigma_{i}=0.7+0.1\cdot i\right\}}_{i\in [1,d]}$	Mild	Ho, $\rho_{\max}=0.85$	Pseudo‐Rank
Model 1.3	Normal	${\left\{\mu_{i}=r\cdot i,\sigma_{i}=0.7+0.1\cdot i\right\}}_{i\in [1,d]}$	Mild	He, $\rho_{\min}=0.05$, $\rho_{\max}=0.35$	Pseudo‐Rank
Model 2.1	Beta	${\left\{\alpha_{i}=2+r\cdot (i-1),\beta_{i}=4-r\cdot (i-1)\right\}}_{i\in [1,d]}$	Mild	None	Pseudo‐Rank
Model 2.2	Beta	${\left\{\alpha_{i}=2+r\cdot (i-1),\beta_{i}=4-r\cdot (i-1)\right\}}_{i\in [1,d]}$	Mild	Ho, $\rho_{\max}=0.85$	Pseudo‐Rank
Model 2.3	Beta	${\left\{\alpha_{i}=2+r\cdot (i-1),\beta_{i}=4-r\cdot (i-1)\right\}}_{i\in [1,d]}$	Mild	He, $\rho_{\min}=0.05$, $\rho_{\max}=0.35$	Pseudo‐Rank
Model 3.1	Poisson	${\left\{\lambda_{i}=5+r\cdot i\right\}}_{i\in [1,d]}$	Mild	None	Pseudo‐Rank
Model 3.2	Poisson	${\left\{\lambda_{i}=5+r\cdot i\right\}}_{i[1,d]}$	Mild	Ho, $\rho_{\max}=0.85$	Pseudo‐Rank
Model 3.3	Poisson	${\left\{\lambda_{i}=5+r\cdot i\right\}}_{i\in [1,d]}$	Mild	He, $\rho_{\min}=0.05$, $\rho_{\max}=0.35$	Pseudo‐Rank

### 7.1 Type‐I Error Rate

Since Table 3 consists of many models of interest, we will present only Models 1.1, 2, 3, and 8.3.1 in this subsection. All the remaining models can be found in Appendix B.1 of the Supporting Information.

Model 1.1 can be referred to as a baseline model. There, the marginal distribution is standard normal, and clusters do not have any within‐correlation. Furthermore, since all marginal distributions are identical, this corresponds to the null hypothesis $H_{0}^{\left(F\right)}$. Additionally, no imbalances were present. We used the pseudo‐rank weighting scheme.

**FIGURE 3:**
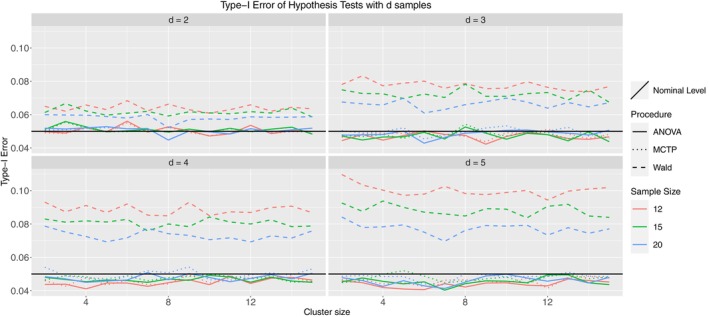
Type‐I error simulation results for Model 1.1; $n_{i}=$ 12 (red), 15 (green), 20 (blue); thick line: ANOVA‐type, dashed line: Wald‐type, dotted line: MCTPs; nominal significance level: α=0.05.

In Figure 3, the Wald‐type test is liberal, whereas the ANOVA‐type and MCTPs control the type‐I error rate very well in all considered scenarios. With an increasing number of groups, both tend to be slightly conservative, but the deviations from the nominal level seem justifiable. Furthermore, increasing sample sizes $n_{i}$ shifts the respective lines of type‐I error rate closer to the nominal level for all tests, whereas the cluster sizes have no visible impact.

**FIGURE 4:**
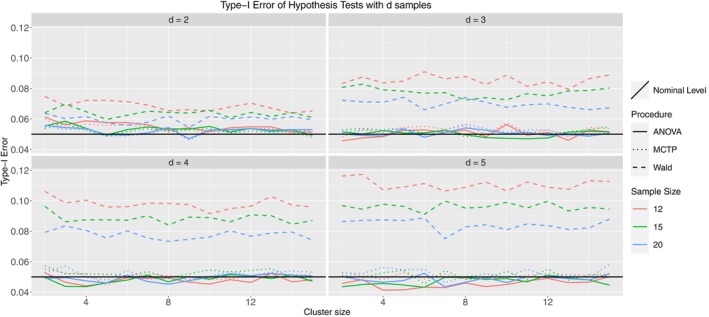
Type‐I error simulation results for Model 2; $n_{i}=$ 12 (red), 15 (green), 20 (blue); thick line: ANOVA‐type, dashed line: Wald‐type, dotted line: MCTPs; nominal significance level: α=0.05.

**FIGURE 5:**
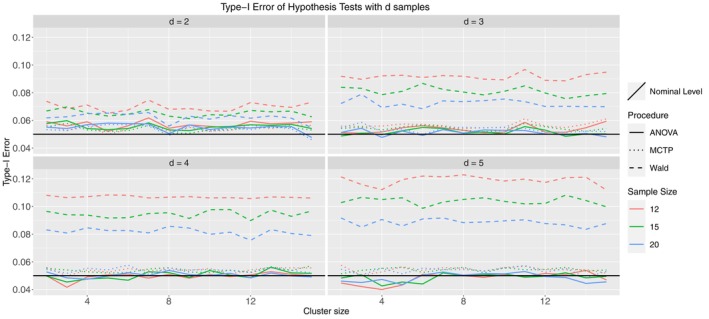
Type‐I error simulation results for Model 3; $n_{i}=$ 12 (red), 15 (green), 20 (blue); thick line: ANOVA‐type, dashed line: Wald‐type, dotted line: MCTP; nominal significance level: α=0.05.

Model 2 differs from Model 1.1 in two ways. First, we induced mild within‐cluster correlation, such that there is a positive association between observations within a single cluster (for all clusters). However, we restricted the correlation to be homogeneous, such that the relationship between observations of a single cluster is identical for all of those observations. Second, we used a beta distribution as the marginal distribution. This is highly relevant for (pre‐)clinical researchers, as this type of distribution is, for example, used in DNA methylation frequency spectra [47]. As to be seen in Figure 4, most of the former results can be confirmed in this model. However, the MCTPs perform slightly better than before, controlling the type‐I error rate well in trials with many groups. As pointed out, the beta distribution is of high practical relevance, implying that our methods provide a well‐suited procedure robust to different data‐generating processes. The next model that we discuss is Model 3. The correlation structure is severe and heterogeneous, meaning that the correlation between two observations within a cluster is not identical among all observations. Furthermore, the marginal distribution was discrete (Poisson). Count data occurs frequently in clinical practice since many endpoints consist of something that is counted (e.g., number of cells, number of proteins, number of injuries). Figure 5 looks like the former one, where the Wald‐type test is too liberal, but the ANOVA‐type test and MCTP control the type‐I error rate well, regardless of the numbers of groups, samples, or cluster sizes.

**FIGURE 6:**
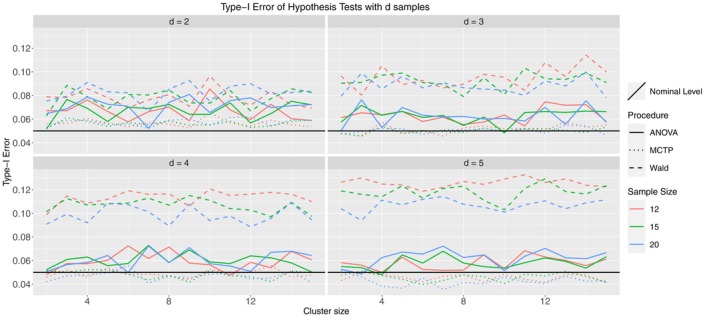
Type‐I error simulation results for Model 8.3.1; $n_{i}=$ 12 (red), 15 (green), 20 (blue); thick line: ANOVA‐type, dashed line: Wald‐type, dotted line: MCTP; nominal significance level: α=0.05.

Finally, Model 8.3.1 is an actual Behrens–Fisher‐type problem. We use the normal distribution centered around 0 as a marginal distribution, but now with different variances across groups. Furthermore, we created a severely imbalanced design, meaning that the sample sizes of the groups differ drastically and that each group contains at least a vast and tiny cluster. Finally, this model includes severe heterogeneous correlation within clusters. From practitioners' point of view, this is a dreadful scenario, as almost every aspect of this model aims to violate a test's validity. However, we want to demonstrate that our proposed methods work, even in a worst‐case scenario. The simulation results are displayed in Figure 6, and we see that the Wald‐type test is very liberal, as in the former models. However, in this scenario, the ANOVA‐type test becomes liberal as well. On the other hand, the MCTP performs very well, especially considering the setting of this model. In the two‐sample case, it is slightly liberal but is roughly within a 1 percentage point deviation from the nominal level. For the 5‐sample case, it is somewhat conservative but also within this deviation. The type‐I error rate seems to be controlled accurately for the 3 and 4 sample cases. As a comparison, Model 8.3.2 (Figure B.1.12 in the Supporting Information) demonstrates the behavior of the same tests in the same settings but using traditional ranks (weighted estimator). Although we prefer pseudo‐ranks, the few models containing the weighted scheme serve as a comparison for the imbalanced designs. One can see that all of the tests tend to be quite liberal, even the MCTP, which underlines the preference for pseudo‐rank procedures. Indeed, the remaining figures in Appendix B of the Supporting Information are very similar to the models presented here. In all cases, the Wald‐type test is too liberal. In most cases, the ANOVA‐type test and MCTP greatly control the type‐I error rate. Furthermore, it is noteworthy that in Models 6.1 (Figure B.1.6 in Supporting Information) and 7 (Figure B.1.8 in Supporting Information), the ANOVA‐type and even the MCTP are liberal for the cases d=2 and d=3. However, they remain far below the type‐I error rate of the Wald‐type test. In Model 6.2 (Figure B.1.7 in Supporting Information), they seem to fail, except for d=5 with large cluster size. This behavior can also be seen in the results for Models 8.2.2 (Figure B.1.11 in Supporting Information) and 8.3.2 (Figure B.1.12 in Supporting Information). All three models have an unbalanced design and use the rank weighting scheme. However, Model 4.2 (Figure B.1.4 in Supporting Information) is also using this weighting scheme but performs considerably well. Given these results, we suspect that the rank estimator can deal well with mild imbalances but cannot deal with severely imbalanced designs. This seems to be a problem for the pseudo‐rank estimator and is noted for Models 6.1 and 7, although it performs better than its rank counterpart. Finally, we also performed the same simulations with a negative correlation instead of a positive correlation. The results are not shown in this work since they are very similar, indicating that the direction of dependency does not matter to the performance of the proposed test procedures.

### 7.2 Type‐II Error Rate

**FIGURE 7:**
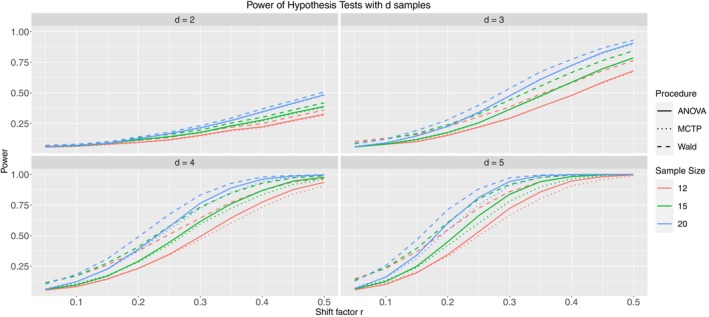
Type‐II error simulation results for Model 1.2; $n_{i}=$ 12 (red), 15 (green), 20 (blue); thick line: ANOVA‐type, dashed line: Wald‐type, dotted line: MCTP; nominal significance level: α=0.05.

**FIGURE 8:**
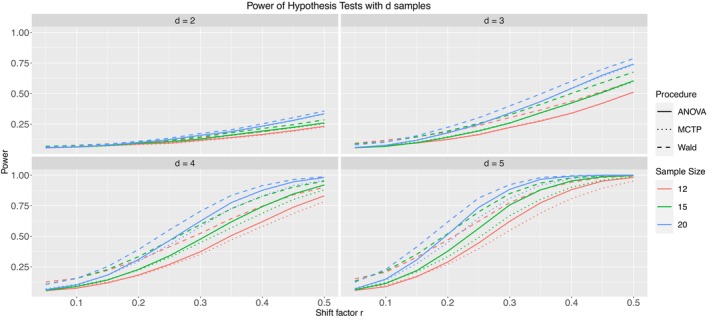
Type‐II error simulation results for Model 2.2; $n_{i}=$ 12 (red), 15 (green), 20 (blue); thick line: ANOVA‐type, dashed line: Wald‐type, dotted line: MCTP; nominal significance level: α=0.05.

**FIGURE 9:**
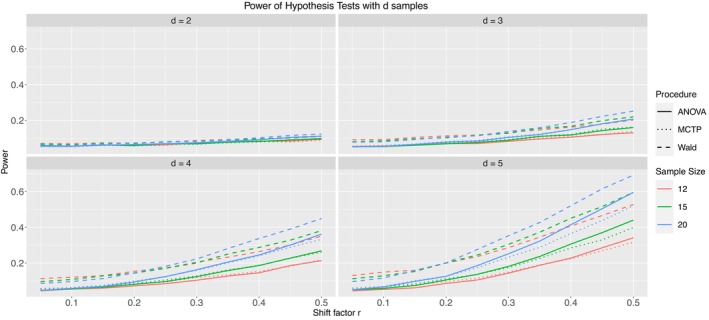
Type‐II error simulation results for Model 3.2; $n_{i}=$ 12 (red), 15 (green), 20 (blue); thick line: ANOVA‐type, dashed line: Wald‐type, dotted line: MCTP; nominal significance level: α=0.05.

Due to the still larger number of models in Table 4, we will only present Models 1.2 (Figure 7), 2.2 (Figure 8), and 3.2 (Figure 9) in this subsection. They include severe but homogeneous correlation within clusters and have mild imbalance among samples and clusters. The remaining results can be found in Appendix B.2 of the Supporting Information. Models 1.1, 2.1, and 3.1 have no intracluster correlation, which is implicitly the same as the increased sample size without clusters. On the other hand, Models 1.3, 2.3, and 3.3 have mild heterogeneous correlations. The pseudo‐rank weighting scheme was applied to all models.

First, we note that with increasing shift factor r and thus increasing effect under the alternative, the power of the test procedures also increases for all models. Furthermore, for a given sample size $n_{i}$, the Wald‐type test shows the highest power. However, this is not surprising since the Wald‐type test also shows inflated type‐I error rates, which necessarily leads to a higher power. Additionally, the ANOVA‐type test and the MCTP tend to have similar power in detecting alternatives, sometimes indistinguishable. The power increases faster with increasing number of groups by definition of our models. The presented models are constructed such that every single group deviates from each other; thus, the total effect grows with an additional group. Furthermore, results not shown within this work indicate that for a given effect, the power does not decrease (and typically increases) with an increasing number of observations per cluster $\left(\frac{\partial (1-\beta )}{\partial m_{ij}}\geq 0\right)$. This can be explained by the idea that additional individual observations add information, although the information gain decreases with increasing correlation. On the other hand, a correlation of 0 is almost identical to additional, independent observations.

## 8 Evaluation of the Real Data Example

This section evaluates the data from the motivating example discussed in Section 2. We use a significance level of α=0.05 and test the null hypothesis $H_{0}:\mathrm{Cp}=0$, where C is a Tukey‐type contrast comparing all groups pair‐wisely, that is,

$$C=\left(\begin{array}{llll} -\;1 & 1 & 0 & 0\\ -\;1 & 0 & 1 & 0\\ -\;1 & 0 & 0 & 1\\ 0 & -1 & 1 & 0\\ 0 & -1 & 0 & 1\\ 0 & 0 & -1 & 1 \end{array}\right)$$

Table 5 summarizes descriptive statistics, namely the relative effects $p_{i}$ for each group using pseudo‐ranks, its standard deviations, and corresponding confidence intervals. It appears that the first group (with smoking status 1) tends to have the highest Berlin grades. On the other hand, the second group tends to have the most minor grades. The remaining groups, 3 and 4, neither tend to have larger or smaller values. However, their standard deviations are quite large, leading to wide confidence intervals.

**TABLE 5:** Estimators and simultaneous confidence intervals of the nonparametric relative effects.

Group	Estimator	Std. Dev	Lower	Upper
1	0.55	0.32	0.43	0.68
2	0.45	0.35	0.32	0.59
3	0.49	0.29	0.38	0.60
4	0.51	0.46	0.33	0.69

Table 6 reports individual contrasts' descriptive statistics and inferential results. For example, the first contrast represents the difference between the first two groups, with their relative effects differing by about 0.1. However, its confidence interval ranges from −0.3 to 0.1, leading to a non‐rejection of the individual null hypothesis. The same conclusion can be drawn from the p value, which is 0.58, hence not significant. Inspecting the other contrasts, none of the individual hypotheses can be rejected on the given significance level. Indeed, the data do not provide the evidence to reject the global null hypothesis using any of the proposed test procedures (p values: Wald‐type: 0.6; ANOVA‐type: 0.66; MCTP: 0.58). We conclude that none of the smoking statuses impacts the Berlin grades at a 5% significance level.

**TABLE 6:** Estimators, simultaneous confidence intervals and p values of the contrasts.

Contrast (groups)	Estimator	Std. Dev	Lower	Upper	p value
2–1	−0.10	0.53	−0.30	0.10	0.58
3–1	−0.06	0.45	−0.24	0.11	0.75
4–1	−0.04	0.67	−0.30	0.22	0.98
3–2	0.03	0.48	−0.15	0.22	0.96
4–2	0.06	0.70	−0.21	0.33	0.93
4–3	0.03	0.64	−0.22	0.27	0.99

## 9 Discussion

Dependent replicates occur frequently, especially in animal testing, laboratory, or social experiments. In statistical practice, data distributions often do not fulfill the assumptions of a (linear) mixed model, making using nonparametric methods necessary. In this article, we develop rank‐based procedures for the nonparametric Behrens–Fisher problem in the presence of (possibly) dependent replicates in general factorial designs. We provide a unified inference framework for estimating relative effects flexibly and testing null hypotheses formulated in terms of relative effects without postulating a specific distributional data model. For completeness, we also develop inference methods for testing rather strict null hypotheses formulated in terms of the distribution functions. The reference distribution and the clusters can be weighed differently and tailored to specific study questions and needs. We recommend using reference distributions that do not rely on sample size allocations, for example, the unweighted effect. Global test procedures and multiple contrast tests for testing null hypotheses have been developed. We focus on approximations of the distributions of the different test statistics in small sample‐size situations. Extensive simulation studies indicate that the developed procedures control the type‐I error level well, even in (very) small sample sizes and heteroscedastic designs. In particular, using a variance stabilizing function (Fisher) improves the behavior of the MCTP [29]. In addition, approximating the distribution of the ANOVA‐type statistic (10) by an F‐distribution significantly improves its behavior. However, the MCTP offers several practical advantages over the ANOVA‐type statistic; individual effects can be reported in addition to the global hypothesis, and the test statistics can be inverted into simultaneous confidence intervals. Especially from the viewpoint of clinical applications, these advantages make the MCTP superior to the ANOVA‐type since individual effects are often of interest, and regulatory authorities require confidence intervals whenever possible.

All of the methods presented are suitable for controlled or homogeneous study environments. None of the methods can account for covariates or other nuisance variables, but future research will include adjustments for covariates. We plan to implement all of the procedures into the R‐package *rankFD* [48] for nonparametric analysis of general factorial designs.

## Conflicts of Interest

The authors declare no conflicts of interest.

## Supporting information




**Appendix S1:** Supporting Information.

**Data S2:** Supporting Information.

## Data Availability

The data that supports the findings of this study are available in the Supporting Information of this article.
