# Supplementary material for: A General Framework for the Multiple Nonparametric Behrens–Fisher Problem With Dependent Replicates
Source: Stat Med. 2024 Nov 11;43(30):5650–66. doi: 10.1002/sim.10262 (PMC11639657; doi:10.1002/sim.10262)
Supplement: Supplementary file 1 — Appendix S1: Supporting Information. [file SIM-43-5650-s002.pdf]

A general framework for the multiple nonparametric Behrens-Fisher problem with dependent replicates

Supplement

This document contains supplementary material of the article *A general framework for the multiple nonparametric Behrens-Fisher problem with dependent replicates* by Sprünken and Konietschke. The first part of this document contains the technical appendix, providing all the proofs that do not appear in the original article. The second part contains additional figures regarding the simulation studies of the article.

Contents

Contents 1

A Technical Details 2
- A.1 Lemma 1 . . . 2
- A.2 Proof - Asymptotic Equivalence . . . 6
- A.3 Proof - Unbiased Covariance Matrix . . . 8
- A.4 Proof - Consistency of the Covariance Matrix . . . 9
- A.5 Proof - Unbiasedness of Variance under $H_0^{(F)}$ . . . 11

B Figures 12
- B.1 Type-I Error Simulation Results . . . 12
- B.2 Type-II Error Simulation Results . . . 18

REFERENCES 21

A | TECHNICAL DETAILS

A.1 | Lemma 1

Let $\mathbf{K} = \int \widehat{F}_{\boldsymbol{\theta}} - F_{\boldsymbol{\theta}} \mathrm{d}(\widehat{\mathbf{F}}^{\psi} - \mathbf{F})$. We state that $\sqrt{g(\mathbf{n})}\mathbf{K} \xrightarrow{\mathcal{L}^2} 0$ under assumptions (A1) - (A6).
Proof: The proof is structured into multiple steps. We will show in (a) that $\mathbb{E}\left[K_i\right] = 0$, in (b) that the non-weight summands of $\mathbb{V}\mathrm{ar}\left(K_i\right)$ converge to some constant $c \in \mathbb{R}$ and in (c) that $\sqrt{g(\mathbf{n})}K_i \in o_p(1)$ as $g(\mathbf{n}) \to \infty$. Finally, the proof is concluded by the fact that $\sqrt{g(\mathbf{n})}K_i \xrightarrow{\mathcal{L}^2} 0\,\forall i \Rightarrow \sqrt{g(\mathbf{n})}\mathbf{K} \xrightarrow{\mathcal{L}^2} 0$.

(a)

$$
\begin{aligned}
K_i &= \int \widehat{F}_{\boldsymbol{\theta}} - F_{\boldsymbol{\theta}} \mathrm{d}(\widehat{F}_i^{\psi} - F_i) \\
&= \int \sum_{i'=1}^{d} \theta_{i'} (\widehat{F}_{i'}^{\psi} - F_{i'}) \mathrm{d}(\widehat{F}_i^{\psi} - F_i) \\
&= \sum_{i'=1}^{d} \theta_{i'} \left(\int \widehat{F}_{i'}^{\psi} - F_{i'} \mathrm{d}(\widehat{F}_i^{\psi} - F_i) \right).
\end{aligned}
$$

Since $\widehat{F}_{i'}^{\psi}$ is an unbiased estimator of $F_{i'}$, the inner term will cancel out in expectation:

$$
\begin{aligned}
\mathbb{E}\left[K_i\right] &= \mathbb{E}\left[\sum_{i'=1}^{d} \theta_{i'} \left(\int \widehat{F}_{i'}^{\psi} - F_{i'} \mathrm{d}(\widehat{F}_i^{\psi} - F_i) \right) \right] \\
&= \sum_{i'=1}^{d} \theta_{i'} \int \mathbb{E}\left[\widehat{F}_{i'}^{\psi} - F_{i'} \right] \mathrm{d}(\widehat{F}_i^{\psi} - F_i).
\end{aligned}
$$

(b)

In this step, we will show that the non-weight components of $\mathbb{V}\mathrm{ar}\left(K_i\right)$ converge to some real constant. Using the result from (a), we only need to compute $\mathbb{V}\mathrm{ar}\left(K_i\right) = \mathbb{E}\left[K_i^2\right]$.

$$
\begin{aligned}
K_i^2 &= \left(\int \widehat{F}_{\boldsymbol{\theta}} - F_{\boldsymbol{\theta}} \mathrm{d}(\widehat{F}_i^{\psi} - F_i) \right) \left(\int \widehat{F}_{\boldsymbol{\theta}} - F_{\boldsymbol{\theta}} \mathrm{d}(\widehat{F}_i^{\psi} - F_i) \right) \\
&= \left(\sum_{i'}^{d} \sum_{j}^{n_{i'}} \theta_{i'} \psi_{i'j} \int \widehat{F}_{i'j}^{\boldsymbol{\omega}} - F_{i'} \mathrm{d}(\widehat{F}_i^{\psi} - F_i) \right) \left(\int \widehat{F}_{\boldsymbol{\theta}} - F_{\boldsymbol{\theta}} \mathrm{d}(\widehat{F}_i^{\psi} - F_i) \right) \\
&= \left(\sum_{i'}^{d} \sum_{j}^{n_{i'}} \theta_{i'} \psi_{i'j} \int \widehat{F}_{i'j}^{\boldsymbol{\omega}} - F_{i'} \mathrm{d}(\sum_{j'}^{n_i} \psi_{ij'} \widehat{F}_{ij'}^{\boldsymbol{\omega}} - F_i) \right) \left(\int \widehat{F}_{\boldsymbol{\theta}} - F_{\boldsymbol{\theta}} \mathrm{d}(\widehat{F}_i^{\psi} - F_i) \right) \\
&= \left(\underbrace{\sum_{i'}^{d} \sum_{j}^{n_{i'}} \sum_{j'}^{n_i} \theta_{i'} \psi_{i'j} \psi_{ij'}}_{:=\sum_{\mathcal{J}}} \int \widehat{F}_{i'j}^{\boldsymbol{\omega}} - F_i \mathrm{d}(\widehat{F}_{ij'}^{\boldsymbol{\omega}} - F_i) \right) \left(\int \widehat{F}_{\boldsymbol{\theta}} - F_{\boldsymbol{\theta}} \mathrm{d}(\widehat{F}_i^{\psi} - F_i) \right) \\
&= \left(\sum_{\mathcal{J}} \int \widehat{F}_{i'j}^{\boldsymbol{\omega}} - F_{i'} \mathrm{d}(\widehat{F}_{ij'}^{\boldsymbol{\omega}} - F_i) \right) \left(\sum_{\mathcal{J}'} \int \widehat{F}_{i''j''}^{\boldsymbol{\omega}} - F_{i''} \mathrm{d}(\widehat{F}_{ij'''}^{\boldsymbol{\omega}} - F_i) \right) \\
&= \sum_{\mathcal{J}} \sum_{\mathcal{J}'} \int \widehat{F}_{i'j}^{\boldsymbol{\omega}} - F_{i'} \mathrm{d}(\widehat{F}_{ij'}^{\boldsymbol{\omega}} - F_i) \int \widehat{F}_{i''j''}^{\boldsymbol{\omega}} - F_{i''} \mathrm{d}(\widehat{F}_{ij'''}^{\boldsymbol{\omega}} - F_i).
\end{aligned}
$$

Now, we have to look at several cases to compute $\mathbb{E}\left[K_i^2\right]$:

1. $i' \neq i''$: In this case we have independence and thus $\mathbb{E}\left[K_i^2\right] = 0$.
2. In case that $i' = i''$ we have in turn two subcases:
   (a) $j \neq j''$ or $j' \neq j'''$: Following the argumentation of Roy et al. (2019) (and here assuming $j = j''$ without loss of generality):

$$\begin{aligned}
&\mathbb{E}\left[\int \widehat{F}^{\omega}_{i'j} - F_{i'} \mathrm{d}(\widehat{F}^{\omega}_{ij'} - F_i) \int \widehat{F}^{\omega}_{i'j} - F_{i'} \mathrm{d}(\widehat{F}^{\omega}_{ij'''} - F_i)\right] \\
= \quad & \mathbb{E}\left[\int \widehat{F}^{\omega}_{i'j} - F_{i'} \mathrm{d}(\widehat{F}^{\omega}_{ij'} - F_i) \mathbb{E}\left[\int \widehat{F}^{\omega}_{i'j} - F_{i'} \mathrm{d}(\widehat{F}^{\omega}_{ij'''} - F_i) \mid \mathbf{X}_{i'j}, \mathbf{X}_{ij'}, \mathbf{X}_{ij'''}\right]\right] \\
= \quad & 0
\end{aligned}$$

   (b) Here, assume that $j = j''$ and $j' = j'''$:

$$\begin{aligned}
&\left|\int \widehat{F}^{\omega}_{i'j} - F_{i'} \mathrm{d}(\widehat{F}^{\omega}_{ij'} - F_i)\right| \\
= \quad & \left|\int \widehat{F}^{\omega}_{i'j} \mathrm{d}\widehat{F}^{\omega}_{ij'} - \int \widehat{F}^{\omega}_{i'j} \mathrm{d}F_i + \int F_{i'} \mathrm{d}F_i - \int F_{i'} \mathrm{d}\widehat{F}^{\omega}_{ij'}\right| \\
\leq \quad & \left|\underbrace{\int \widehat{F}^{\omega}_{i'j} \mathrm{d}\widehat{F}^{\omega}_{ij'}}_{\max 1} - \underbrace{\int \widehat{F}^{\omega}_{i'j} \mathrm{d}F_i}_{\min 0}\right| + \left|\underbrace{\int F_{i'} \mathrm{d}F_i}_{\max 1} - \underbrace{\int F_{i'} \mathrm{d}\widehat{F}^{\omega}_{ij'}}_{\min 0}\right| \\
\leq \quad & 2 \\
\Rightarrow & \int \widehat{F}^{\omega}_{i'j} - F_{i'} \mathrm{d}(\widehat{F}^{\omega}_{ij'} - F_i) \int \widehat{F}^{\omega}_{i'j} - F_{i'} \mathrm{d}(\widehat{F}^{\omega}_{ij'} - F_i) \quad \leq \quad 4.
\end{aligned}$$

   The first inequality used here is the triangle inequality.

(c)

Finally, to compute $\mathbb{E}\left[K_i^2\right]$, we know from (b) that only the summands from $j = j''$ and at the same time $j' = j'''$ are relevant. Furthermore, we know from (b) that the product of integrals is less than some constant. Thus, we will ignore this constant and only have a look at the sums of weights in these cases and their relation to $g(\mathbf{n})$.

First, look at the double sum over the index sets $\mathcal{J}$ and $\mathcal{J}'$.

$$
\begin{aligned}
&\sum_{\mathcal{J}}\sum_{\mathcal{J}'} \mathbb{1}\left(i'=i''\right)\mathbb{1}\left(j=j''\right)\mathbb{1}\left(j'=j'''\right) \\
=\;& \sum_{i'}^{d}\sum_{j}^{n_{i'}}\sum_{j'}^{n_i}\sum_{i''}^{d}\sum_{j''}^{n_{i''}}\sum_{j'''}^{n_i} \mathbb{1}\left(i'=i''\right)\mathbb{1}\left(j=j''\right)\mathbb{1}\left(j'=j'''\right)\psi_{i'j}\psi_{ij'}\psi_{i''j''}\psi_{ij'''}\theta_{i'}\theta_{i''} \\
=\;& \sum_{i'}^{d}\sum_{j}^{n_{i'}}\sum_{i''}^{d}\sum_{j''}^{n_{i''}}\theta_{i'}\theta_{i''}\mathbb{1}\left(i'=i''\right)\mathbb{1}\left(j=j''\right)\psi_{i'j}\psi_{i''j''}\sum_{j'}^{n_i}\sum_{j'''}^{n_i}\mathbb{1}\left(j'=j'''\right)\psi_{ij'}\psi_{ij'''} \\
=\;& \sum_{i'}^{d}\sum_{i''}^{d}\mathbb{1}\left(i'=i''\right)\theta_{i'}\theta_{i''}\sum_{j}^{n_{i'}}\sum_{j''}^{n_{i''}}\mathbb{1}\left(j=j''\right)\psi_{i'j}\psi_{i''j''}\sum_{j'}^{n_i}\sum_{j'''}^{n_i}\psi_{ij'}\psi_{ij'''}\mathbb{1}\left(j'=j'''\right) \\
=\;& \sum_{i'}^{d}\theta_{i'}^2\sum_{j}^{n_{i'}}\sum_{j''}^{n_{i''}}\mathbb{1}\left(j=j''\right)\psi_{i'j}\psi_{i''j''}\sum_{j'}^{n_i}\sum_{j'''}^{n_i}\psi_{ij'}\psi_{ij'''}\mathbb{1}\left(j'=j'''\right) \\
=\;& \sum_{i'}^{d}\theta_{i'}^2\sum_{j}^{n_{i'}}\psi_{i'j}^2\sum_{j'}^{n_i}\sum_{j'''}^{n_i}\psi_{ij'}\psi_{ij'''}\mathbb{1}\left(j'=j'''\right) \\
=\;& \sum_{i'}^{d}\theta_{i'}^2\sum_{j}^{n_{i'}}\psi_{i'j}^2\sum_{j'}^{n_i}\psi_{ij'}^2 \\
=\;& \sum_{j'}^{n_i}\psi_{ij'}^2\sum_{i'}^{d}\theta_{i'}^2\sum_{j}^{n_{i'}}\psi_{i'j}^2.
\end{aligned}
$$

Thus, including $g(\mathbf{n})$, we have:

$$
g(\mathbf{n})\mathbb{E}\left[K_i^2\right] = g(\mathbf{n})\sum_{j'}^{n_i}\psi_{ij'}^2\sum_{i'}^{d}\theta_{i'}^2\sum_{j}^{n_{i'}}\psi_{i'j}^2.
$$

By assumption, $g(\mathbf{n})\psi_{ij} \to c \;\forall i,j$, thus:

$$
\begin{aligned}
g(\mathbf{n})\mathbb{E}\left[K_i^2\right] &= \sum_{j'}^{n_i}\psi_{ij'}^2\sum_{i'}^{d}\theta_{i'}^2\sum_{j}^{n_{i'}}g(\mathbf{n})\psi_{i'j}^2 \\
&= \sum_{j'}^{n_i}\psi_{ij'}^2\sum_{i'}^{d}\theta_{i'}^2\sum_{j}^{n_{i'}}c\psi_{i'j} \\
&\le c\sum_{j'}^{n_i}\psi_{ij'}^2\sum_{i'}^{d}\theta_{i'}^2\sum_{j}^{n_{i'}}\psi_{i'j} \\
&\le c\sum_{j'}^{n_i}\psi_{ij'}^2\sum_{i'}^{d}\theta_{i'}^2.
\end{aligned}
$$

Now, for $z_i \in \mathbb{R}^+, i \in (1, ..., n)$ we know that:

$$
\sum_{i=1}^{n} z_i^2 \le \left(\sum_{i=1}^{n} z_i\right)^2.
$$

Thus:

$$
\begin{aligned}
\sum_{j'}^{n_i}\psi_{ij'}^2\sum_{i'}^{d}\theta_{i'}^2 &\le \sum_{j'}^{n_i}\psi_{ij'}^2\left(\sum_{i'}^{d}\theta_{i'}\right)^2 \\
&\le \sum_{j'}^{n_i}\psi_{ij'}^2\cdot 1^2
\end{aligned}
$$

Thus, under the aforementioned assumption that $\psi_{ij} \to 0 \; \forall i, j$ as $n \to \infty$, the term above converges to 0. Thus, we conclude that $\sqrt{g(\mathbf{n})} K_i \xrightarrow{\mathcal{L}^2} 0$ and as a result that $\sqrt{g(\mathbf{n})} K_i \in o_p(1)$.

A.2 | Proof - Asymptotic Equivalence

Proof of Theorem 1: Note, that we can factorize the term $g(\mathbf{n})$, thus we have:

$$\left\|\sqrt{g(\mathbf{n})}\left((\widehat{\mathbf{p}}-\mathbf{p})-\left(1-2\mathbf{p}+\sum_{i=1}^{d}\sum_{j=1}^{n_i}\psi_{ij}\mathbf{A}_{ij}\right)\right)\right\|_2^2 \quad = \quad g(\mathbf{n})\left\|(\widehat{\mathbf{p}}-\mathbf{p})-\left(1-2\mathbf{p}+\sum_{i=1}^{d}\sum_{j=1}^{n_i}\psi_{ij}\mathbf{A}_{ij}\right)\right\|_2^2$$

At first, we conduct some algebra including the use of empty sums:

$$\begin{aligned}
\widehat{\mathbf{p}}-\mathbf{p} &= \int \widehat{F}_{\boldsymbol{\theta}}\mathrm{d}\widehat{\mathbf{F}}^{\psi} - \int F_{\boldsymbol{\theta}}\mathrm{d}\mathbf{F} \\
&= \int \widehat{F}_{\boldsymbol{\theta}}\mathrm{d}\widehat{\mathbf{F}}^{\psi} - \underbrace{\int F_{\boldsymbol{\theta}}\mathrm{d}\mathbf{F}}_{\mathbf{p}} + \left(\underbrace{\int F_{\boldsymbol{\theta}}\mathrm{d}\mathbf{F}}_{\mathbf{p}} - \underbrace{\int F_{\boldsymbol{\theta}}\mathrm{d}\mathbf{F}}_{\mathbf{p}}\right) + \left(\underbrace{\int \widehat{F}_{\boldsymbol{\theta}}\mathrm{d}\mathbf{F}}_{1-\int \mathbf{F}\mathrm{d}\widehat{F}_{\boldsymbol{\theta}}} - \underbrace{\int \widehat{F}_{\boldsymbol{\theta}}\mathrm{d}\mathbf{F}}_{1-\int \mathbf{F}\mathrm{d}\widehat{F}_{\boldsymbol{\theta}}}\right) + \left(\int F_{\boldsymbol{\theta}}\mathrm{d}\widehat{\mathbf{F}}^{\psi} - \int F_{\boldsymbol{\theta}}\mathrm{d}\widehat{\mathbf{F}}^{\psi}\right) \\
&= \int F_{\boldsymbol{\theta}}\mathrm{d}\widehat{\mathbf{F}}^{\psi} - \int \mathbf{F}\mathrm{d}\widehat{F}_{\boldsymbol{\theta}} + 1 - 2\mathbf{p} + \left(\underbrace{\int \widehat{F}_{\boldsymbol{\theta}}\mathrm{d}\widehat{\mathbf{F}}^{\psi} - \int \widehat{F}_{\boldsymbol{\theta}}\mathrm{d}\mathbf{F} - \int F_{\boldsymbol{\theta}}\mathrm{d}\widehat{\mathbf{F}}^{\psi} + \int F_{\boldsymbol{\theta}}\mathrm{d}\mathbf{F}}_{\int \widehat{F}_{\boldsymbol{\theta}}-F_{\boldsymbol{\theta}}\mathrm{d}(\widehat{\mathbf{F}}^{\psi}-\mathbf{F})=\mathbf{K}}\right) \\
&= \int F_{\boldsymbol{\theta}}\mathrm{d}\widehat{\mathbf{F}}^{\psi} - \int \mathbf{F}\mathrm{d}\widehat{F}_{\boldsymbol{\theta}} + 1 - 2\mathbf{p} + \mathbf{K}.
\end{aligned}$$

In a second step, we will show that $\int F_{\boldsymbol{\theta}}\mathrm{d}\widehat{\mathbf{F}}^{\psi} - \int \mathbf{F}\mathrm{d}\widehat{F}_{\boldsymbol{\theta}}$ can be represented by $\sum_{i=1}^{d}\sum_{j=1}^{n_i}\psi_{ij}\mathbf{A}_{ij}$. For simplicity, consider only the h-th group and effect here:

$$\begin{aligned}
\int F_{\boldsymbol{\theta}}\mathrm{d}\widehat{F}_h - \int F_h\mathrm{d}\widehat{F}_{\boldsymbol{\theta}} &= \sum_{j=1}^{n_h}\psi_{hj}\sum_{k=1}^{m_{hj}}\omega_{hjk}F_{\boldsymbol{\theta}} - \sum_{i=1}^{d}\sum_{j=1}^{n_i}\psi_{ij}\theta_i\underbrace{\sum_{k=1}^{m_{ij}}\omega_{ijk}F_h(X_{ijk})}_{:=Y_{ij}^{(h)}} \\
&= \sum_{j=1}^{n_h}\psi_{hj}\sum_{k=1}^{m_{hj}}\omega_{hjk}F_{\boldsymbol{\theta}} - \sum_{i=1}^{d}\sum_{j=1}^{n_i}\psi_{ij}\theta_i Y_{ij}^{(h)} \\
&= \sum_{j=1}^{n_h}\psi_{hj}\sum_{k=1}^{m_{hj}}\omega_{hjk}\left(\sum_{i=1}^{d}\theta_i F_i(X_{hjk})\right) - \sum_{i=1}^{d}\sum_{j=1}^{n_i}\psi_{ij}\theta_i Y_{ij}^{(h)} \\
&= \sum_{i=1}^{d}\theta_i\sum_{j=1}^{n_h}\psi_{hj}\underbrace{\sum_{k=1}^{m_{hj}}\omega_{hjk}F_i(X_{hjk})}_{:=Y_{hj}^{(i)}} - \sum_{i=1}^{d}\sum_{j=1}^{n_i}\psi_{ij}\theta_i Y_{ij}^{(h)} \\
&= \sum_{i=1}^{d}\theta_i\sum_{j=1}^{n_h}\psi_{hj}Y_{hj}^{(i)} - \sum_{i=1}^{d}\sum_{j=1}^{n_i}\psi_{ij}\theta_i Y_{ij}^{(h)} \\
&= \sum_{\substack{i=1\\ i\neq h}}^{d}\theta_i\sum_{j=1}^{n_h}\psi_{hj}Y_{hj}^{(i)} + \theta_h\sum_{j=1}^{n_h}\psi_{hj}Y_{hj}^{(h)} \\
&\quad - \left(\sum_{\substack{i=1\\ i\neq h}}^{d}\sum_{j=1}^{n_i}\psi_{ij}\theta_i Y_{ij}^{(h)} + \theta_h\sum_{j=1}^{n_h}\psi_{hj}Y_{hj}^{(h)}\right) \\
&= \sum_{\substack{i=1\\ i\neq h}}^{d}\theta_i\sum_{j=1}^{n_h}\psi_{hj}Y_{hj}^{(i)} - \sum_{\substack{i=1\\ i\neq h}}^{d}\sum_{j=1}^{n_i}\psi_{ij}\theta_i Y_{ij}^{(h)}.
\end{aligned}$$

The following should be noted here: The index i of the first double sum refers to the distribution function used within Y, whereas the index i of the second double sum refers only to the sample of the observations used within Y. To avoid further confusion, we will use the index s instead of i for the first sum:

$$\int F_{\boldsymbol{\theta}} \mathrm{d}\widehat{F}_h - \int F_h \mathrm{d}\widehat{F}_{\boldsymbol{\theta}} = \sum_{\substack{s=1\\ s\neq h}}^{d} \theta_s \sum_{j=1}^{n_h} \psi_{hj} Y_{hj}^{(s)} - \sum_{\substack{i=1\\ i\neq h}}^{d} \sum_{j=1}^{n_i} \psi_{ij}\theta_i Y_{ij}^{(h)},$$

and note that we could rewrite a single term as a summand over itself, such as $z_b = \sum_{i=b}^{b} z_i$. It follows that the former equation can be rewritten as follows:

$$\sum_{\substack{s=1\\ s\neq h}}^{d} \theta_s \sum_{j=1}^{n_h} \psi_{hj} Y_{hj}^{(s)} - \sum_{\substack{i=1\\ i\neq h}}^{d} \sum_{j=1}^{n_i} \psi_{ij}\theta_i Y_{ij}^{(h)} = \sum_{i=h}^{h} \sum_{j=1}^{n_i} \psi_{ij} \sum_{\substack{s=1\\ s\neq i}}^{d} \theta_s Y_{ij}^{(s)} - \sum_{\substack{i=1\\ i\neq h}}^{d} \sum_{j=1}^{n_i} \psi_{ij}\theta_i Y_{ij}^{(h)}.$$

Finally, we see that these are two sums over two complements of the same index set $\mathcal{I}$. Thus we define:

$$A_{hij} = \begin{cases} \sum_{\substack{s=1\\ s\neq i}}^{d} \theta_s Y_{ij}^{(s)} & h = i \\ -\theta_i Y_{ij}^{(h)} & h \neq i. \end{cases},$$

and can thus rewrite:

$$\sum_{i=h}^{h} \sum_{\substack{s=1\\ s\neq i}}^{d} \theta_s \sum_{j=1}^{n_i} \psi_{ij} Y_{ij}^{(s)} - \sum_{\substack{i=1\\ i\neq h}}^{d} \sum_{j=1}^{n_i} \psi_{ij}\theta_i Y_{ij}^{(h)} = \sum_{i=1}^{d} \sum_{j=1}^{n_i} \psi_{ij} A_{hij}.$$

Finally, to complete the proof of the $\mathcal{L}^2$-Convergence, note that we have shown

$$\widehat{\mathbf{p}} - \mathbf{p} = 1 - 2\mathbf{p} + \sum_{i=1}^{d} \sum_{j=1}^{n_i} \psi_{ij} \mathbf{A}_{ij} + \mathbf{K},$$

and that $\sqrt{g(\mathbf{n})}\mathbf{K} \xrightarrow{\mathcal{L}^2} 0$ by Lemma 1 in Appendix A.1.

A.3 Proof - Unbiased Covariance Matrix

The estimator

$$\widehat{\boldsymbol{\Sigma}}^* = g(\mathbf{n}) \sum_{i=1}^{d} \sum_{j=1}^{n_i} \psi_{ij}^2 \kappa_{ij}^{-1} \widehat{\boldsymbol{\Sigma}}_{ij}^*$$

is an unbiased estimator of $\boldsymbol{\Sigma}$. **Proof:** For notational purposes, let $\mathbb{V}\text{ar}\left(\mathbf{A}_{ij}\right) = \boldsymbol{\Sigma}_i$. First, take the expectation of the estimator $\widehat{\boldsymbol{\Sigma}}^*$:

$$\begin{aligned}
\mathbb{E}\left[\widehat{\boldsymbol{\Sigma}}^*\right] &= \mathbb{E}\left[g(\mathbf{n}) \sum_{i=1}^{d} \sum_{j=1}^{n_i} \psi_{ij}^2 \widehat{\boldsymbol{\Sigma}}_{ij}^*\right] \\
&= g(\mathbf{n}) \sum_{i=1}^{d} \sum_{j=1}^{n_i} \psi_{ij}^2 \mathbb{E}\left[\widehat{\boldsymbol{\Sigma}}_{ij}^*\right].
\end{aligned}$$

Thus, the expectation of the estimator depends on the expectation of $\widehat{\boldsymbol{\Sigma}}_{ij}^*$. This leads to the following lines:

$$\begin{aligned}
\mathbb{E}\left[\widehat{\boldsymbol{\Sigma}}_{ij}^*\right] &= \mathbb{E}\left[\mathbf{A}_{ij}\mathbf{A}_{ij}^\intercal\right] - \mathbb{C}\text{ov}\left(\overline{\mathbf{A}}_{i\bullet}^*, \mathbf{A}_{ij}\right) - \mathbb{C}\text{ov}\left(\mathbf{A}_{ij}, \overline{\mathbf{A}}_{i\bullet}^*\right) \\
&\quad - 2\mathbb{E}\left[\mathbf{A}_{ij}\right]\mathbb{E}\left[\mathbf{A}_{ij}\right]^\intercal + \mathbb{E}\left[\overline{\mathbf{A}}_{i\bullet}^* \overline{\mathbf{A}}_{i\bullet}^{*\intercal}\right] \\
&= \mathbb{E}\left[\mathbf{A}_{ij}\mathbf{A}_{ij}^\intercal\right] - \psi_{ij}\mathbb{C}\text{ov}\left(\mathbf{A}_{ij}, \mathbf{A}_{ij}\right) - \sum_{j' \neq j}^{n_i} \psi_{ij'}\mathbb{C}\text{ov}\left(\mathbf{A}_{ij'}, \mathbf{A}_{ij}\right) \\
&\quad - \psi_{ij}\mathbb{C}\text{ov}\left(\mathbf{A}_{ij}, \mathbf{A}_{ij}\right) - \sum_{j' \neq j}^{n_i} \psi_{ij'}\mathbb{C}\text{ov}\left(\mathbf{A}_{ij}, \mathbf{A}_{ij'}\right) - 2\mathbb{E}\left[\mathbf{A}_{ij}\right]\mathbb{E}\left[\mathbf{A}_{ij}\right]^\intercal + \mathbb{E}\left[\overline{\mathbf{A}}_{i\bullet}^* \overline{\mathbf{A}}_{i\bullet}^{*\intercal}\right] \\
&= \mathbb{E}\left[\mathbf{A}_{ij}\mathbf{A}_{ij}^\intercal\right] - 2\psi_{ij}\mathbb{V}\text{ar}\left(\mathbf{A}_{ij}\right) - 2\mathbb{E}\left[\mathbf{A}_{ij}\right]\mathbb{E}\left[\mathbf{A}_{ij}\right]^\intercal + \mathbb{E}\left[\overline{\mathbf{A}}_{i\bullet}^* \overline{\mathbf{A}}_{i\bullet}^{*\intercal}\right] \\
&= \mathbb{E}\left[\mathbf{A}_{ij}\mathbf{A}_{ij}^\intercal\right] - 2\psi_{ij}\boldsymbol{\Sigma}_i - 2\mathbb{E}\left[\mathbf{A}_{ij}\right]\mathbb{E}\left[\mathbf{A}_{ij}\right]^\intercal + \mathbb{E}\left[\overline{\mathbf{A}}_{i\bullet}^* \overline{\mathbf{A}}_{i\bullet}^{*\intercal}\right] \\
&= \boldsymbol{\Sigma}_i + \mathbb{E}\left[\mathbf{A}_{ij}\right]\mathbb{E}\left[\mathbf{A}_{ij}\right]^\intercal - 2\psi_{ij}\boldsymbol{\Sigma}_i - 2\mathbb{E}\left[\mathbf{A}_{ij}\right]\mathbb{E}\left[\mathbf{A}_{ij}\right]^\intercal \\
&\quad + \sum_{j'=1}^{n_i} \psi_{ij}^2 \boldsymbol{\Sigma}_i + \mathbb{E}\left[\overline{\mathbf{A}}_{i\bullet}^*\right]\mathbb{E}\left[\overline{\mathbf{A}}_{i\bullet}^{*\intercal}\right] \\
&= \boldsymbol{\Sigma}_i - 2\psi_{ij}\boldsymbol{\Sigma}_i + \boldsymbol{\Sigma}_i \sum_{j'=1}^{n_i} \psi_{ij'}^2 \\
&= \boldsymbol{\Sigma}_i \left(1 - 2\psi_{ij} + \sum_{j'=1}^{n_i} \psi_{ij'}^2\right) \\
&= \boldsymbol{\Sigma}_i \kappa_{ij}
\end{aligned}$$

A.4 | Proof - Consistency of the Covariance Matrix

Proof of Theorem 3: Since the estimator is asymptotically unbiased, it suffices to show that $\mathbb{V}\text{ar}\left(\widehat{\boldsymbol{\Sigma}}^*\right) \to 0$ as $g(\mathbf{n}) \to \infty$ and that the estimator $\widehat{\boldsymbol{\Sigma}}$ is close enough to $\widehat{\boldsymbol{\Sigma}}^*$. Using the Theorem from Popoviciu (1935), the term $\widehat{\boldsymbol{\Sigma}}^*_{ij}$ is bounded.[1] Since the variance of the term above must be bounded too, the only interest lies in the limiting behavior of the weights and the bias correction. The following equation shows that we can rearrange the term in such a way that there is no further dependence on n.

$$
\begin{aligned}
\mathbb{V}\text{ar}\left(\widehat{\boldsymbol{\Sigma}}^*\right) &= \mathbb{V}\text{ar}\left(g(\mathbf{n})\sum_{i=1}^{d}\sum_{j=1}^{n_i}\psi_{ij}^2\kappa_{ij}^{-1}\widehat{\boldsymbol{\Sigma}}^*_{ij}\right)\\
&= g(\mathbf{n})^2\mathbb{V}\text{ar}\left(\sum_{i=1}^{d}\sum_{j=1}^{n_i}\psi_{ij}^2\kappa_{ij}^{-1}\widehat{\boldsymbol{\Sigma}}^*_{ij}\right)\\
&= g(\mathbf{n})^2\sum_{i=1}^{d}\sum_{j=1}^{n_i}\psi_{ij}^4\kappa_{ij}^{-2}\mathbb{V}\text{ar}\left(\widehat{\boldsymbol{\Sigma}}^*_{ij}\right)\\
&\le g(\mathbf{n})^2\sum_{i=1}^{d}\sum_{j=1}^{n_i}\psi_{ij}^4\kappa_{ij}^{-2}c_{ij}.
\end{aligned}
$$

Note, that due to the theorem cited above $c_{ij} \in \mathbb{R}^+$ is a bounded constant which does not depend on n.
By assumption, $\kappa_{ij}^2 \to 1$ and $\psi_{ij}^4 \to 0$, such that the unbiased and unobservable variance estimator converges in $\mathcal{L}^2$ to the true variance.

Now, we want to show that the observable variance estimator is sufficiently close to the unobservable one. To do so, we show $\mathcal{L}^2$ convergence of the observable estimator concerning the unobservable estimator.

$$
\begin{aligned}
\mathbb{E}\left[\left(\widehat{\boldsymbol{\Sigma}}-\widehat{\boldsymbol{\Sigma}}^*\right)^2\right] &= \mathbb{E}\left[\left(g(\mathbf{n})\sum_{i=1}^{d}\sum_{j=1}^{n_i}\psi_{ij}^2\kappa_{ij}^{-1}\widehat{\boldsymbol{\Sigma}}_{ij} - g(\mathbf{n})\sum_{i=1}^{d}\sum_{j=1}^{n_i}\psi_{ij}^2\kappa_{ij}^{-1}\widehat{\boldsymbol{\Sigma}}^*_{ij}\right)^2\right]\\
&= \mathbb{E}\left[\left(g(\mathbf{n})\sum_{i=1}^{d}\sum_{j=1}^{n_i}\psi_{ij}^2\kappa_{ij}^{-1}\left(\widehat{\boldsymbol{\Sigma}}_{ij}-\widehat{\boldsymbol{\Sigma}}^*_{ij}\right)\right)^2\right]\\
&= g(\mathbf{n})^2\mathbb{E}\left[\left(\sum_{i=1}^{d}\sum_{j=1}^{n_i}\psi_{ij}^2\kappa_{ij}^{-1}\left(\widehat{\boldsymbol{\Sigma}}_{ij}-\widehat{\boldsymbol{\Sigma}}^*_{ij}\right)\right)^2\right]
\end{aligned}
$$

In the following, we will deal with elementwise $\mathcal{L}^2$ convergence. For readability, we will rewrite the weighted mean of the observable variance estimator:

$$
\widehat{\bar{\mathbf{A}}}_{i\bullet} = \sum_{j=1}^{n_i}\psi_{ij}\widehat{\mathbf{A}}_{ij}.
$$

Since the unobservable estimator is computed as an empirical variance-covariance matrix

$$
\left(\mathbf{A}_{ij}-\bar{\mathbf{A}}_{i\bullet}\right)\left(\mathbf{A}_{ij}-\bar{\mathbf{A}}_{i\bullet}\right)^{\mathsf{T}},
$$

the k-th diagonal entry has the form $\left(A_{kij} - \bar{A}_{ki\cdot}\right)^2$ and the observable estimator follows: $\left(\widehat{A}_{kij} - \widehat{\bar{A}}_{ki\cdot}\right)^2$. Therefore:

$$\begin{aligned}
\mathbb{E}\left[\left(\widehat{\boldsymbol{\Sigma}} - \widehat{\boldsymbol{\Sigma}}^*\right)^2\right] &= g(\mathbf{n})^2 \mathbb{E}\left[\left(\sum_{i=1}^{d}\sum_{j=1}^{n_i} \psi_{ij}^2 \kappa_{ij}^{-1}\left(\widehat{\boldsymbol{\Sigma}}_{ij} - \widehat{\boldsymbol{\Sigma}}_{ij}^*\right)\right)^2\right] \\
&= g(\mathbf{n})^2 \mathbb{E}\left[\left(\sum_{i=1}^{d}\sum_{j=1}^{n_i} \psi_{ij}^2 \kappa_{ij}^{-1} \underbrace{\left(\left(\widehat{A}_{kij} - \widehat{\bar{A}}_{ki\cdot}\right)^2 - \left(A_{kij} - \bar{A}_{ki\cdot}\right)^2\right)}_{:=B_{ij}}\right)^2\right].
\end{aligned}$$

Since both squares in the inner brackets are bounded for all i, j, we can rewrite (with $B_{max} = \max_{i,j} B_{ij}$):

$$\begin{aligned}
\mathbb{E}\left[\left(\widehat{\boldsymbol{\Sigma}} - \widehat{\boldsymbol{\Sigma}}^*\right)^2\right] &\leq g(\mathbf{n})^2 \mathbb{E}\left[\left(\sum_{i=1}^{d}\sum_{j=1}^{n_i} \psi_{ij}^2 \kappa_{ij}^{-1} B_{max}\right)^2\right] \\
&\leq g(\mathbf{n})^2 \left(B_{max} \sum_{i=1}^{d}\sum_{j=1}^{n_i} \psi_{ij}^2 \kappa_{ij}^{-1}\right)^2 \\
&\leq g(\mathbf{n})^2 B_{max}^2 \left(\sum_{i,j} \psi_{ij}^2 \kappa_{ij}^{-1}\right)^2 \\
&\leq g(\mathbf{n})^2 B_{max}^2 \left(\sum_{i,j} (\psi_{ij}^2)^2\right)\left(\sum_{i,j} (\kappa_{ij}^{-1})^2\right).
\end{aligned}$$

The penultimate line is Fubini and the last line Cauchy-Schwartz. Finally:

$$\begin{aligned}
g(\mathbf{n})^2 B_{max}^2 \left(\sum_{i,j} (\psi_{ij}^2)^2\right)\left(\sum_{i,j} (\kappa_{ij}^{-1})^2\right) &\leq g(\mathbf{n})^2 B^2 \left(\sum_{i,j} \psi_{ij}^4\right)\left(\sum_{i,j} \kappa_{ij}^{-2}\right) \\
&\leq B_{max}^2 \left(\sum_{i,j} \left(g(\mathbf{n})\psi_{ij}^2\right)^2\right)\left(\sum_{i,j} \kappa_{ij}^{-2}\right) \\
&\leq B_{max}^2 \left(\sum_{i,j} (c\ \psi_{ij})^2\right)\left(\sum_{i,j} \kappa_{ij}^{-2}\right) \\
&\leq B_{max}^2 \left(c^2 \sum_{i,j} \psi_{ij}^2\right)\left(\sum_{i,j} \kappa_{ij}^{-2}\right).
\end{aligned}$$

The penultimate line here relies on the assumption that $g(\mathbf{n}) \cdot \psi_{ij} \to c\ \forall i, j$. By assumption, $\psi_{ij}^2 \to 0$ and $\kappa_{ij}^{-2} \to 1$, thus the whole product converges to 0.
The proof for the off-diagonal elements follows similar manipulations and arguments, and thus is omitted here.

A.5 | Proof - Unbiasedness of Variance under $H_0^{(F)}$

Proof: The proof follows the same computation as Proof A.3 in Appendix A.3, with the only difference that this proof is concerned with scalars instead of vectors. Thus, some lines are skipped.

$$
\begin{aligned}
\mathbb{E}\left[\widehat{\sigma}_i^{*2}\right] &= \sum_{j=1}^{n_i} \psi_{ij}^2 \mathbb{E}\left[\left(Y_{ij} - \overline{Y}_{i\cdot}\right)^2\right] \\
&= \sum_{j=1}^{n_i} \psi_{ij}^2 \mathbb{E}\left[Y_{ij}^2 - 2Y_{ij}\overline{Y}_{i\cdot}^{*} + \overline{Y}_{i\cdot}^{*2}\right] \\
&= \sum_{j=1}^{n_i} \psi_{ij}^2 \left(\sigma_{ij}^2 + \mathbb{E}\left[Y_{ij}\right]^2 - 2\psi_{ij}\sigma_{ij}^2 + 2\mathbb{E}\left[Y_{ij}\right]^2 + \sigma_{ij}^2 \sum_{j'=1}^{n_i} \psi_{ij'}^2 + \mathbb{E}\left[Y_{ij}\right]^2\right) \\
&= \sum_{j=1}^{n_i} \psi_{ij}^2 \sigma_{ij}^2 \underbrace{\left(1 - 2\psi_{ij} + \sum_{j'=1}^{n_i} \psi_{ij'}^2\right)}_{:=\kappa_{ij}}
\end{aligned}
$$

B | FIGURES

B.1 | Type-I Error Simulation Results

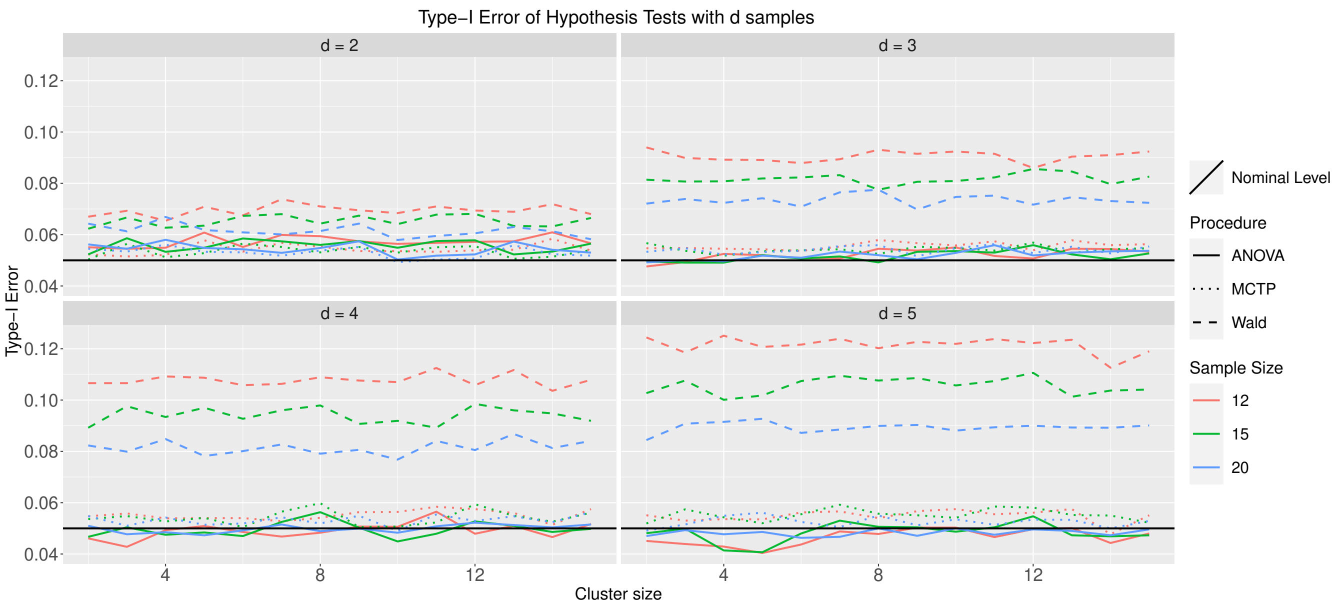

FIGURE B.1.1 Type-I Error Simulation Results for Model 1.2; $n_i = 12$ (red), 15 (green), 20 (blue); Thick Line: ANOVA-type, Dashed Line: Wald-type, Dotted Line: MCTP; Nominal significance level: $\alpha = 0.05$

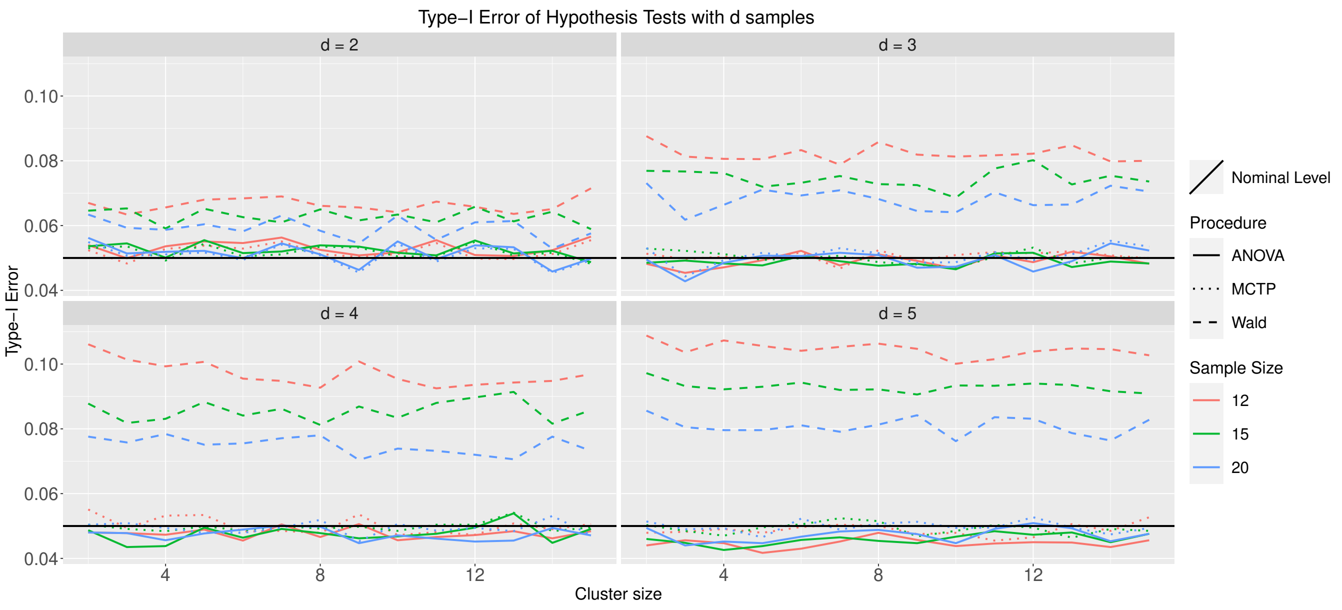

FIGURE B.1.2 Type-I Error Simulation Results for Model 1.3; $n_i = 12$ (red), 15 (green), 20 (blue); Thick Line: ANOVA-type, Dashed Line: Wald-type, Dotted Line: MCTP; Nominal significance level: $\alpha = 0.05$

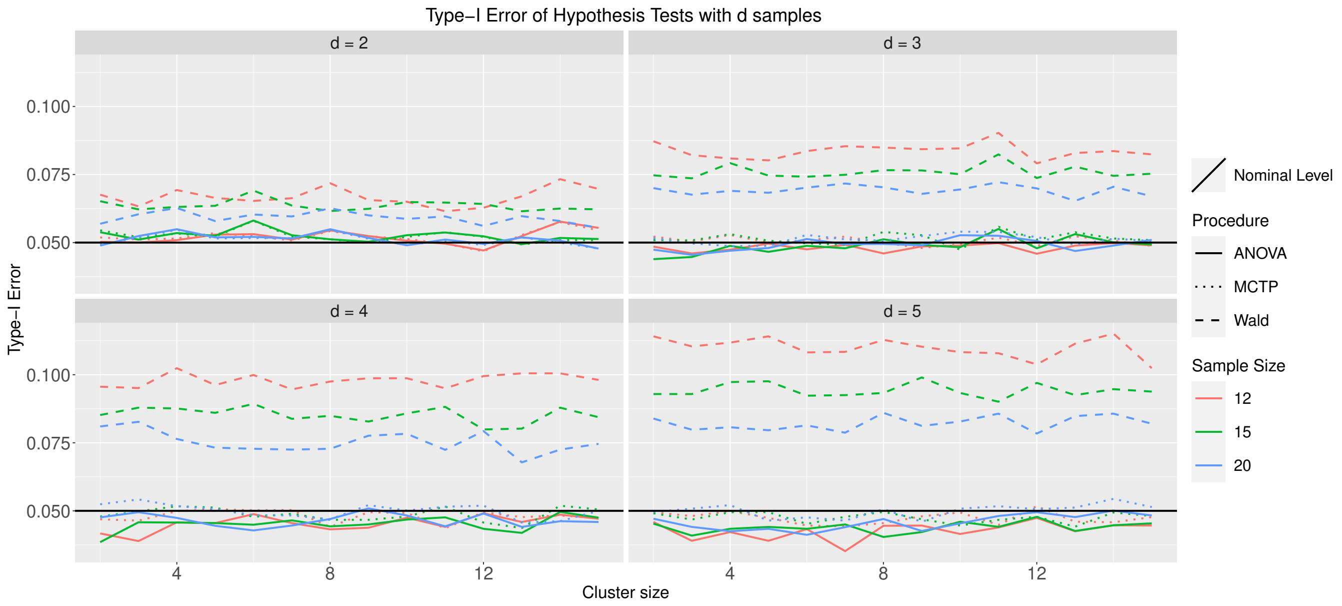

FIGURE B.1.3 Type-I Error Simulation Results for Model 4.1; n_i = 12 (red), 15 (green), 20 (blue); Thick Line: ANOVA-type, Dashed Line: Wald-type, Dotted Line: MCTP; Nominal significance level: $\alpha = 0.05$

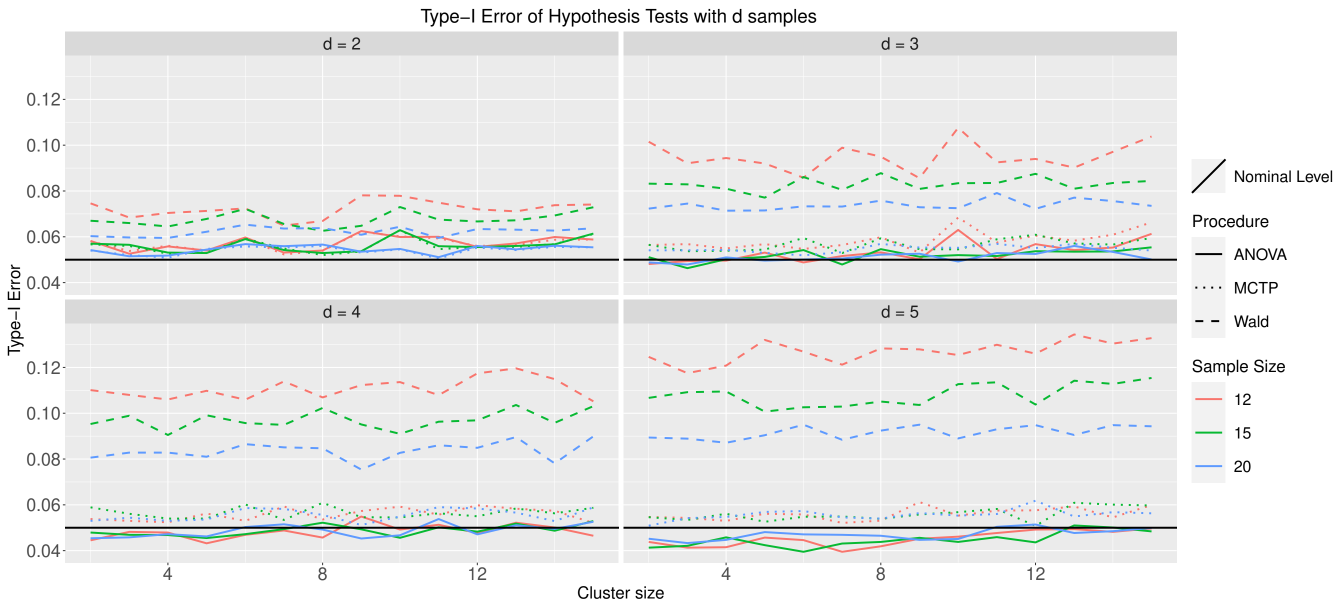

FIGURE B.1.4 Type-I Error Simulation Results for Model 4.2; n_i = 12 (red), 15 (green), 20 (blue); Thick Line: ANOVA-type, Dashed Line: Wald-type, Dotted Line: MCTP; Nominal significance level: $\alpha = 0.05$

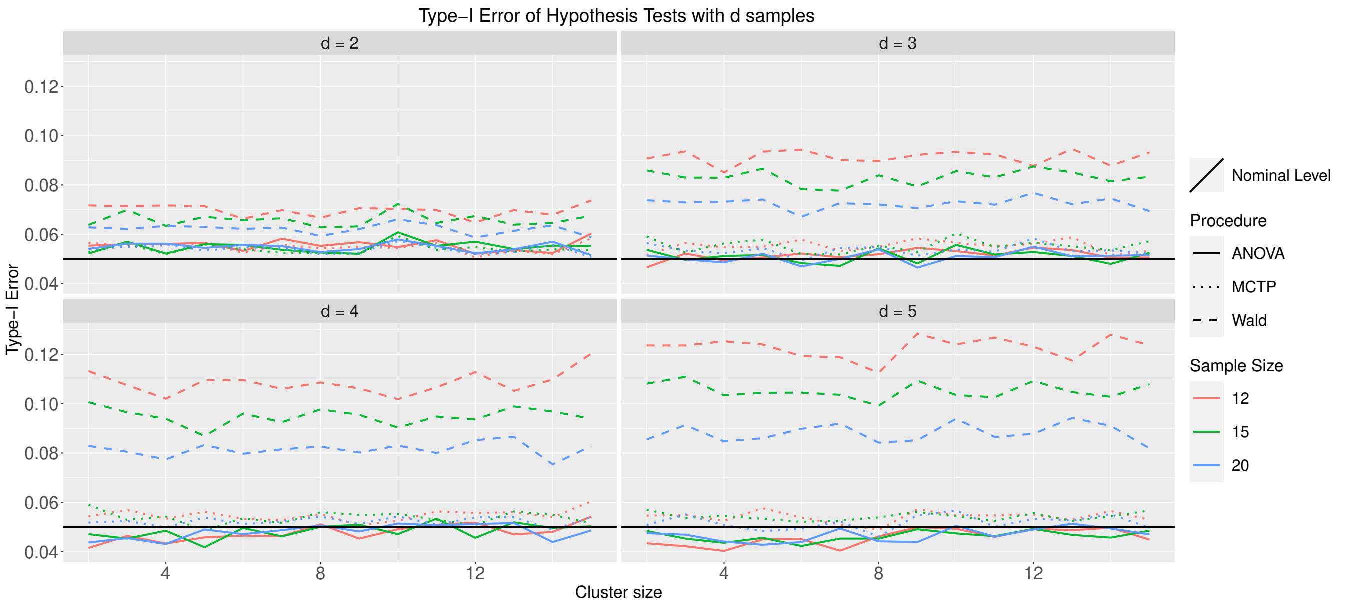

FIGURE B.1.5 Type-I Error Simulation Results for Model 5; n_i = 12 (red), 15 (green), 20 (blue); Thick Line: ANOVA-type, Dashed Line: Wald-type, Dotted Line: MCTP; Nominal significance level: $\alpha = 0.05$

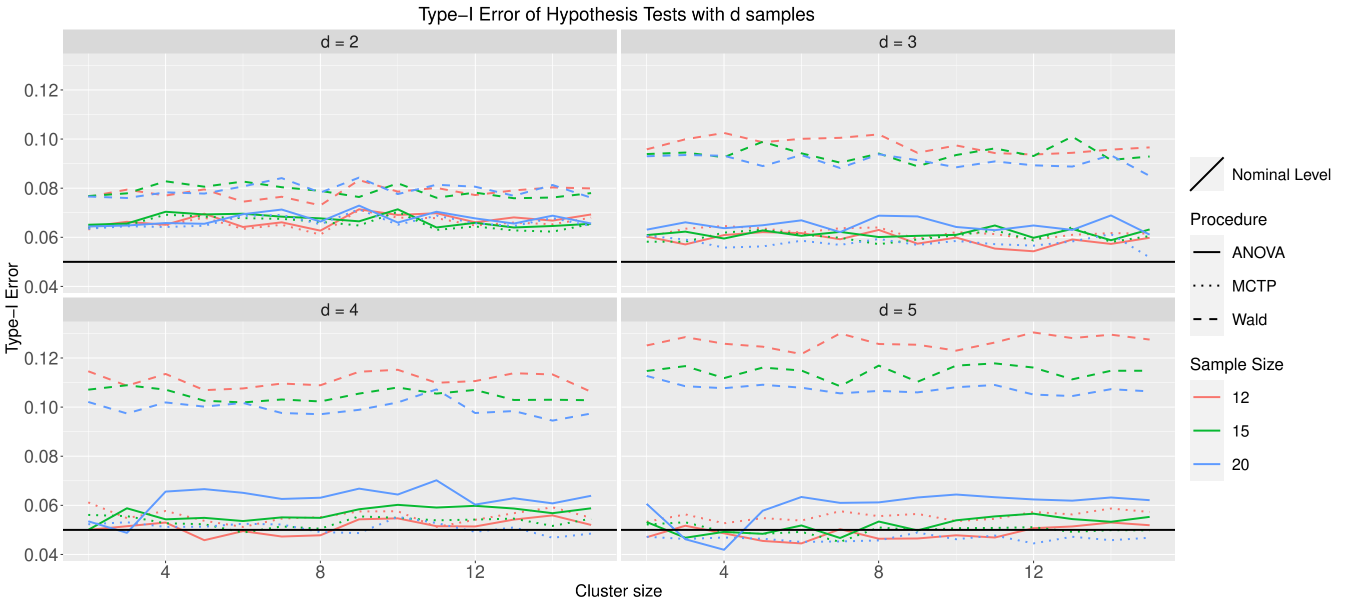

FIGURE B.1.6 Type-I Error Simulation Results for Model 6.1; n_i = 12 (red), 15 (green), 20 (blue); Thick Line: ANOVA-type, Dashed Line: Wald-type, Dotted Line: MCTP; Nominal significance level: $\alpha = 0.05$

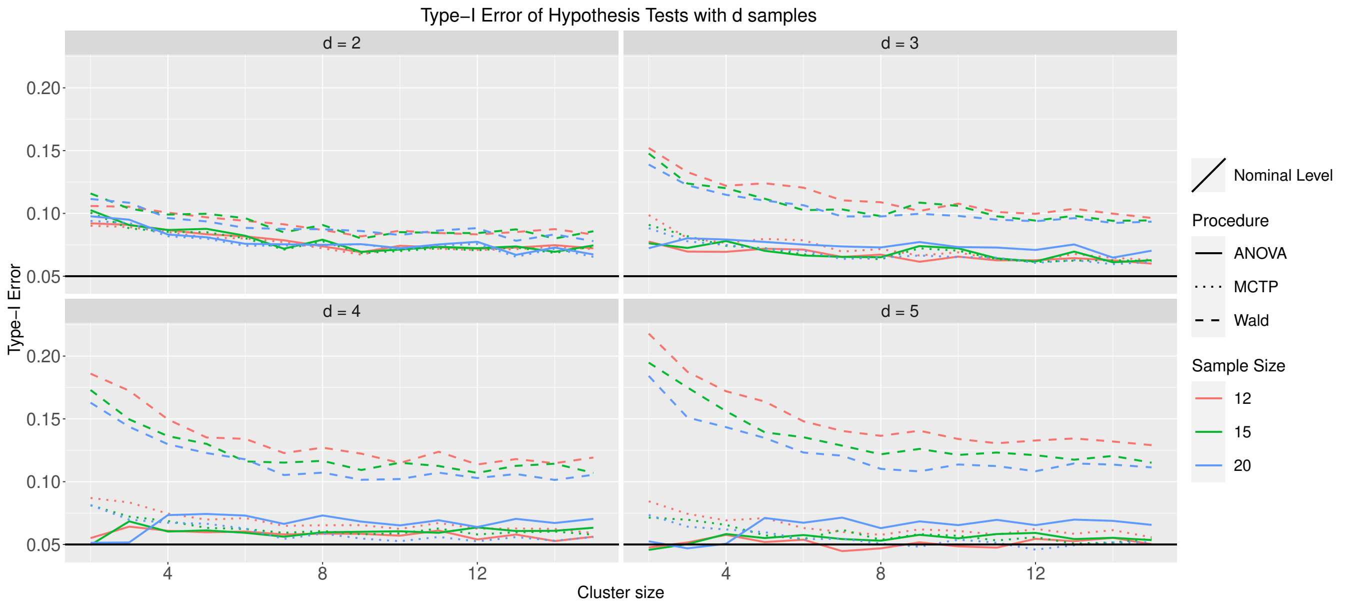

FIGURE B.1.7 Type-I Error Simulation Results for Model 6.2; n_i = 12 (red), 15 (green), 20 (blue); Thick Line: ANOVA-type, Dashed Line: Wald-type, Dotted Line: MCTP; Nominal significance level: α = 0.05

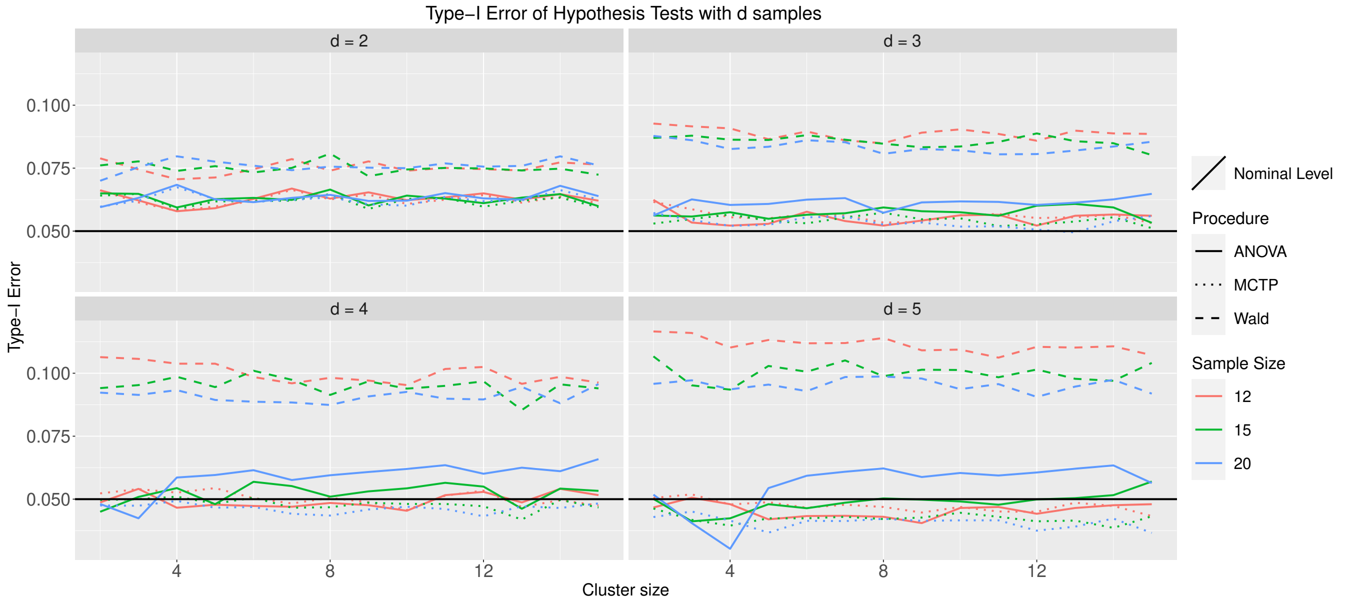

FIGURE B.1.8 Type-I Error Simulation Results for Model 7; n_i = 12 (red), 15 (green), 20 (blue); Thick Line: ANOVA-type, Dashed Line: Wald-type, Dotted Line: MCTP; Nominal significance level: α = 0.05

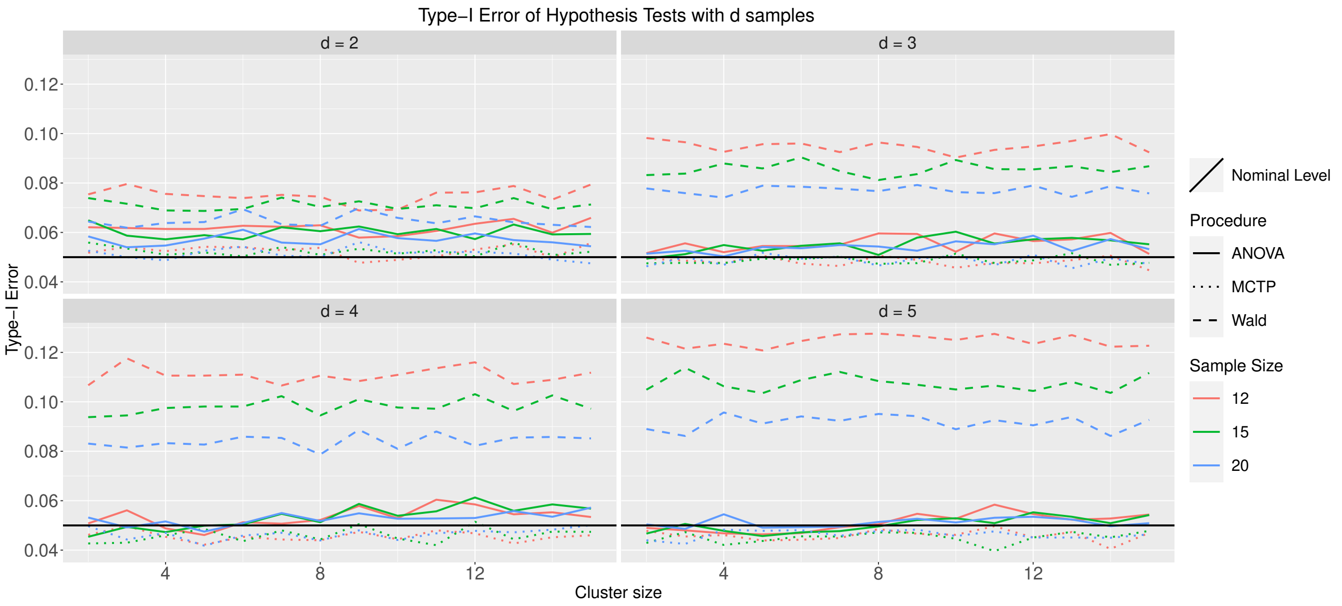

FIGURE B.1.9 Type-I Error Simulation Results for Model 8.1; n_i = 12 (red), 15 (green), 20 (blue); Thick Line: ANOVA-type, Dashed Line: Wald-type, Dotted Line: MCTP; Nominal significance level: α = 0.05

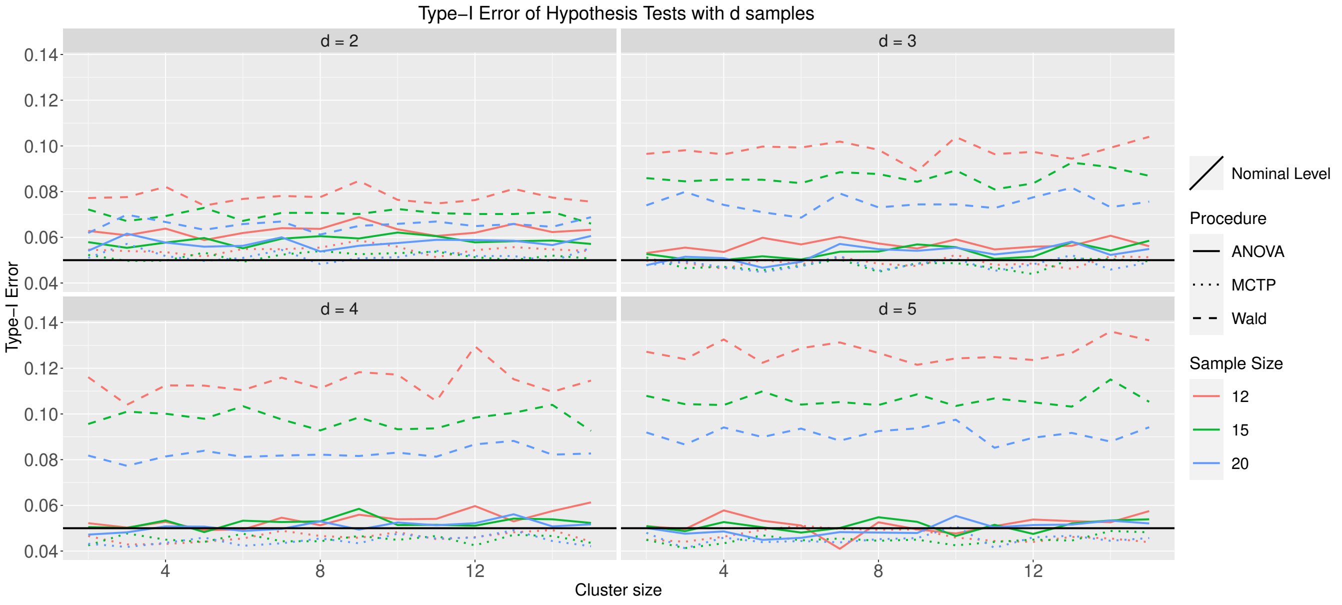

FIGURE B.1.10 Type-I Error Simulation Results for Model 8.2.1; n_i = 12 (red), 15 (green), 20 (blue); Thick Line: ANOVA-type, Dashed Line: Wald-type, Dotted Line: MCTP; Nominal significance level: α = 0.05

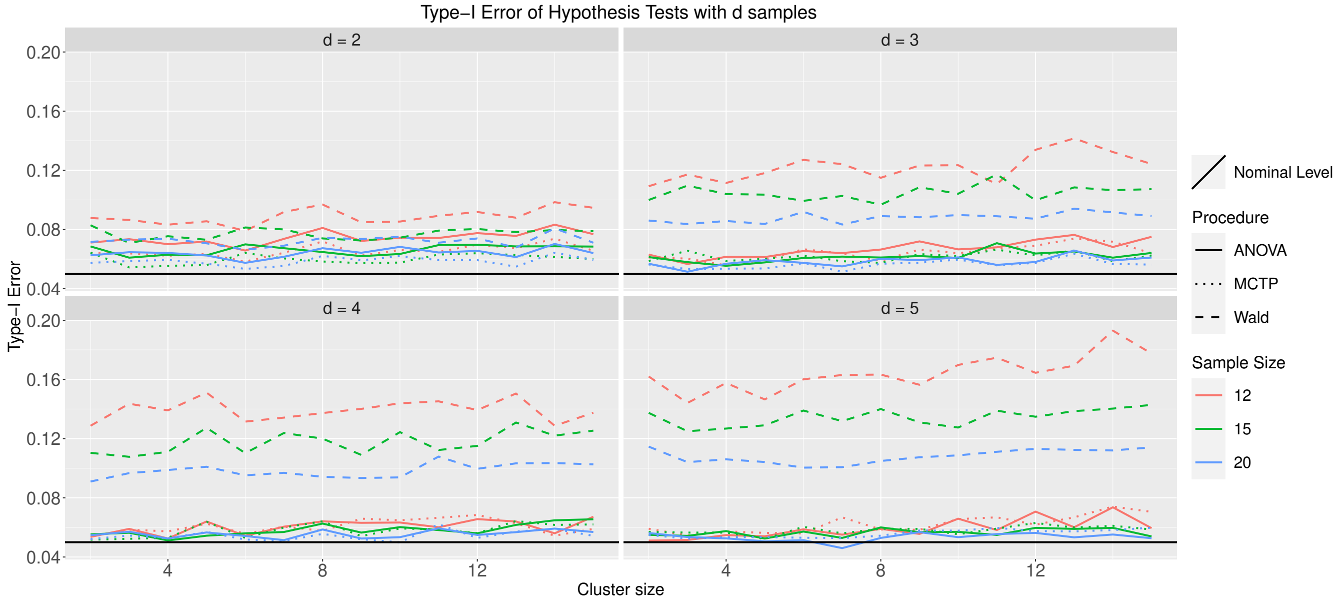

FIGURE B.1.11 Type-I Error Simulation Results for Model 8.2.2; n_i = 12 (red), 15 (green), 20 (blue); Thick Line: ANOVA-type, Dashed Line: Wald-type, Dotted Line: MCTP; Nominal significance level: $\alpha = 0.05$

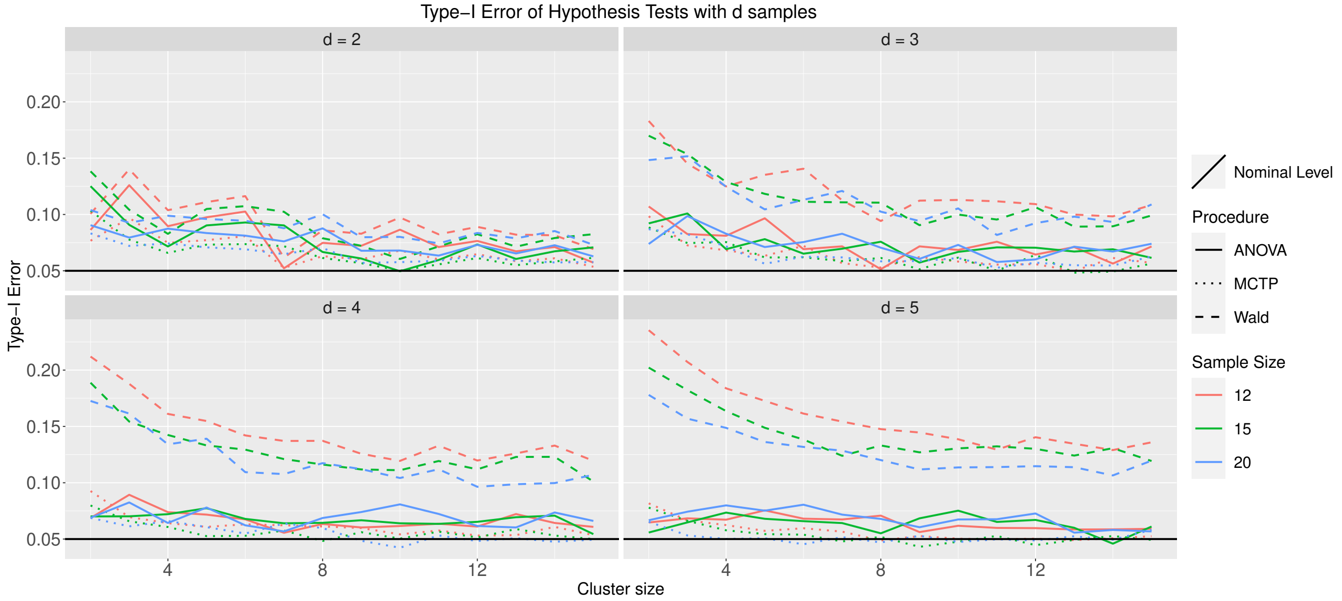

FIGURE B.1.12 Type-I Error Simulation Results for Model 8.3.2; n_i = 12 (red), 15 (green), 20 (blue); Thick Line: ANOVA-type, Dashed Line: Wald-type, Dotted Line: MCTP; Nominal significance level: $\alpha = 0.05$

B.2 | Type-II Error Simulation Results

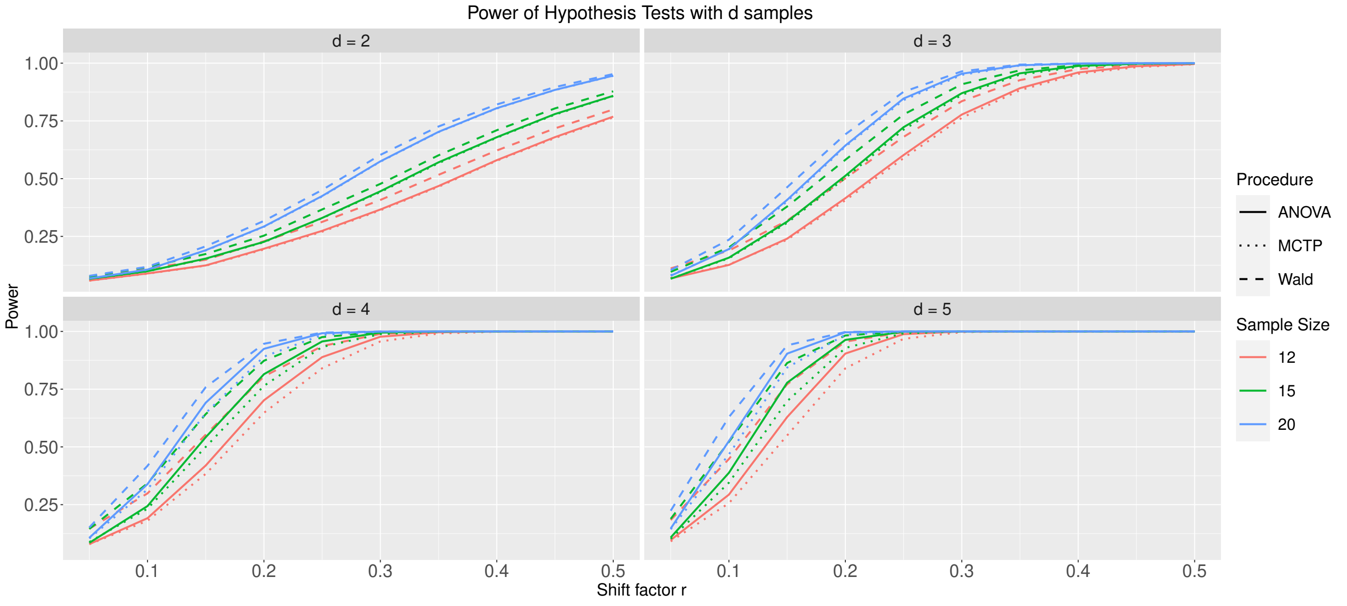

FIGURE B.2.1 Type-II Error Simulation Results for Model 1.1; n_i = 12 (red), 15 (green), 20 (blue); Thick Line: ANOVA-type, Dashed Line: Wald-type, Dotted Line: MCTP; Nominal significance level: $\alpha = 0.05$

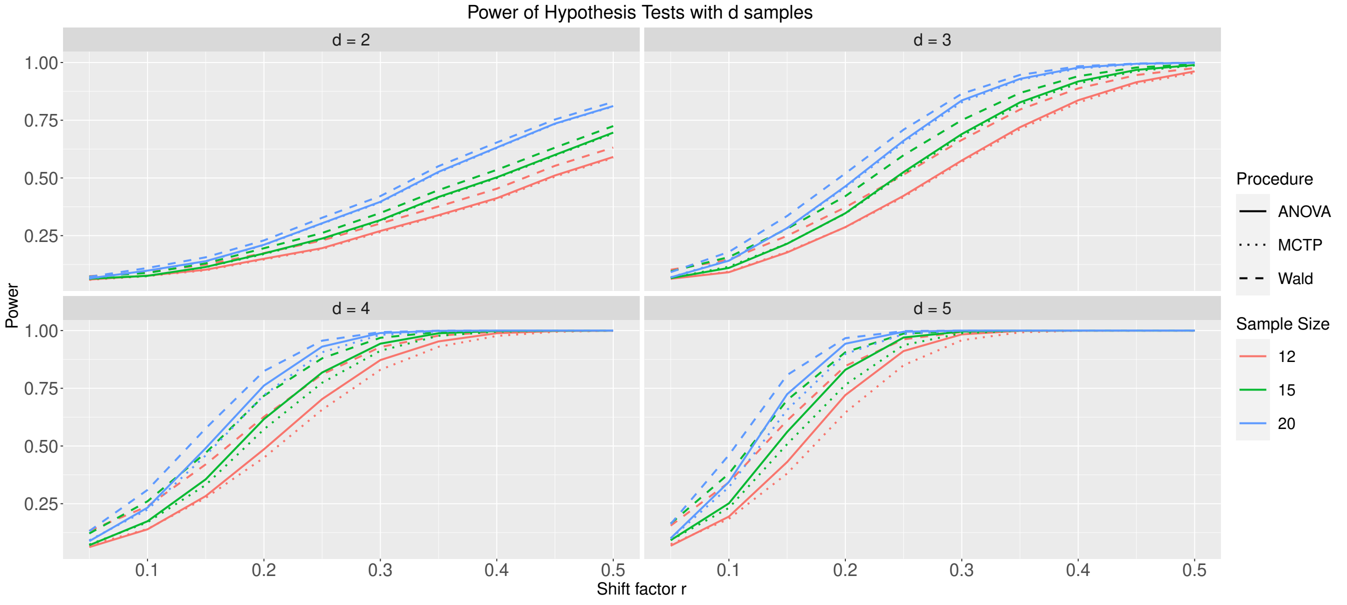

FIGURE B.2.2 Type-II Error Simulation Results for Model 1.3; n_i = 12 (red), 15 (green), 20 (blue); Thick Line: ANOVA-type, Dashed Line: Wald-type, Dotted Line: MCTP; Nominal significance level: $\alpha = 0.05$

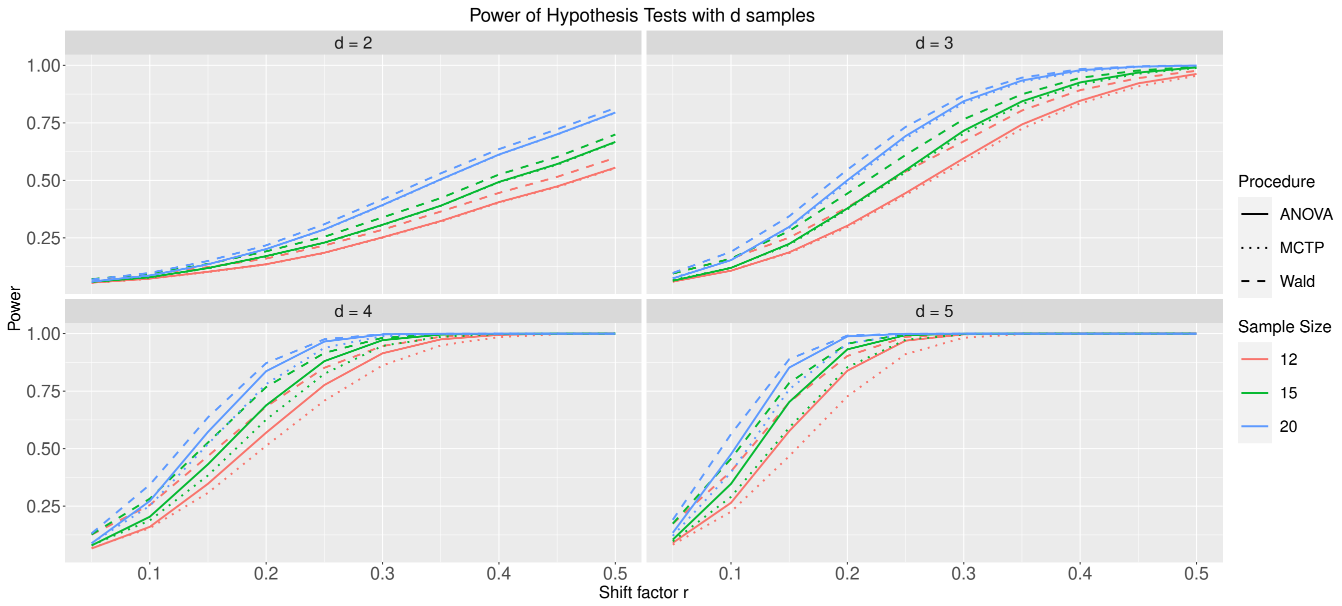

FIGURE B.2.3 Type-II Error Simulation Results for Model 2.1; n_i = 12 (red), 15 (green), 20 (blue); Thick Line: ANOVA-type, Dashed Line: Wald-type, Dotted Line: MCTP; Nominal significance level: $\alpha = 0.05$

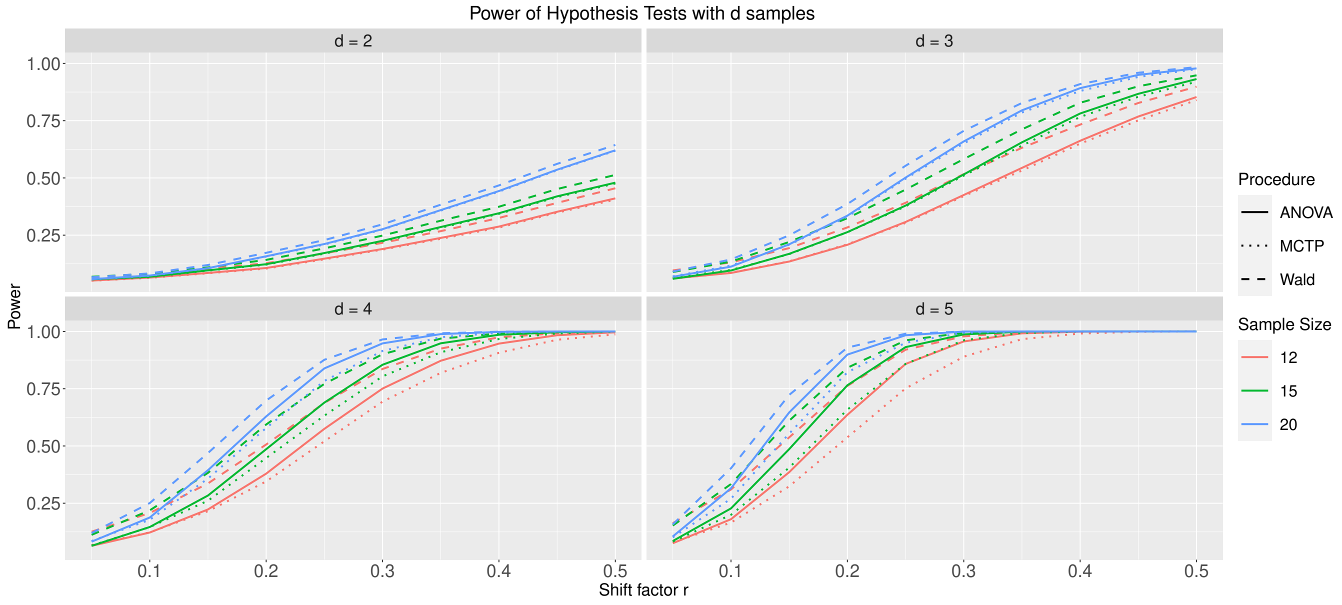

FIGURE B.2.4 Type-II Error Simulation Results for Model 2.3; n_i = 12 (red), 15 (green), 20 (blue); Thick Line: ANOVA-type, Dashed Line: Wald-type, Dotted Line: MCTP; Nominal significance level: $\alpha = 0.05$

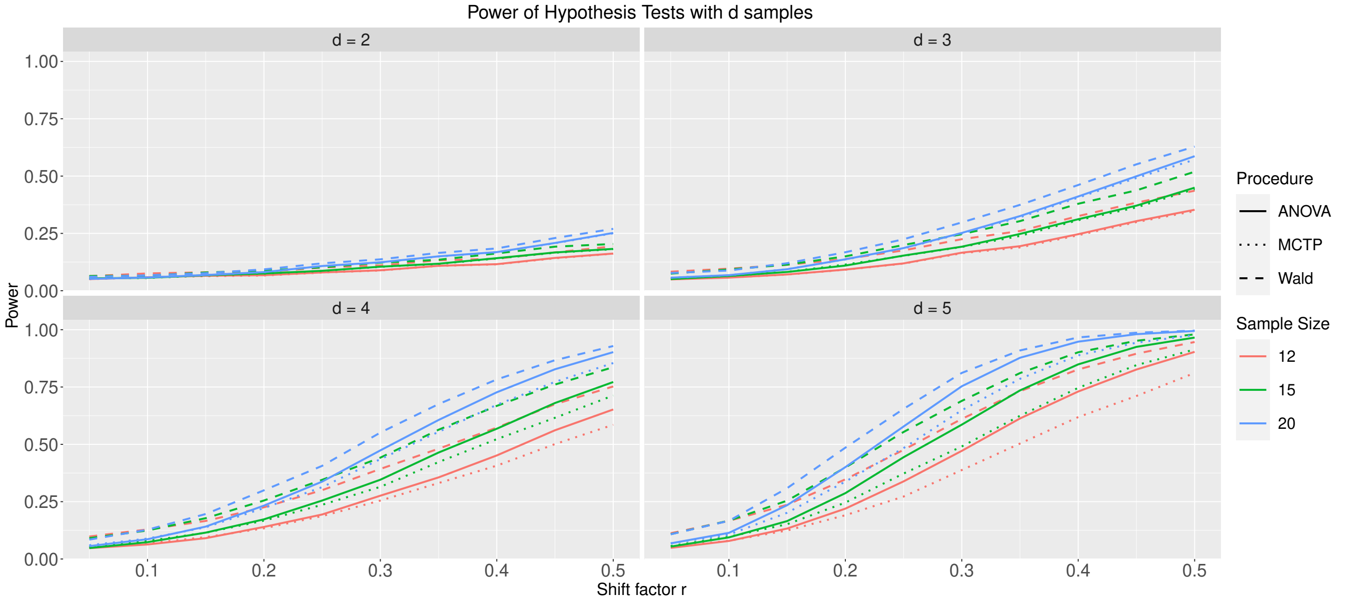

FIGURE B.2.5 Type-II Error Simulation Results for Model 3.1; n_i = 12 (red), 15 (green), 20 (blue); Thick Line: ANOVA-type, Dashed Line: Wald-type, Dotted Line: MCTP; Nominal significance level: α = 0.05

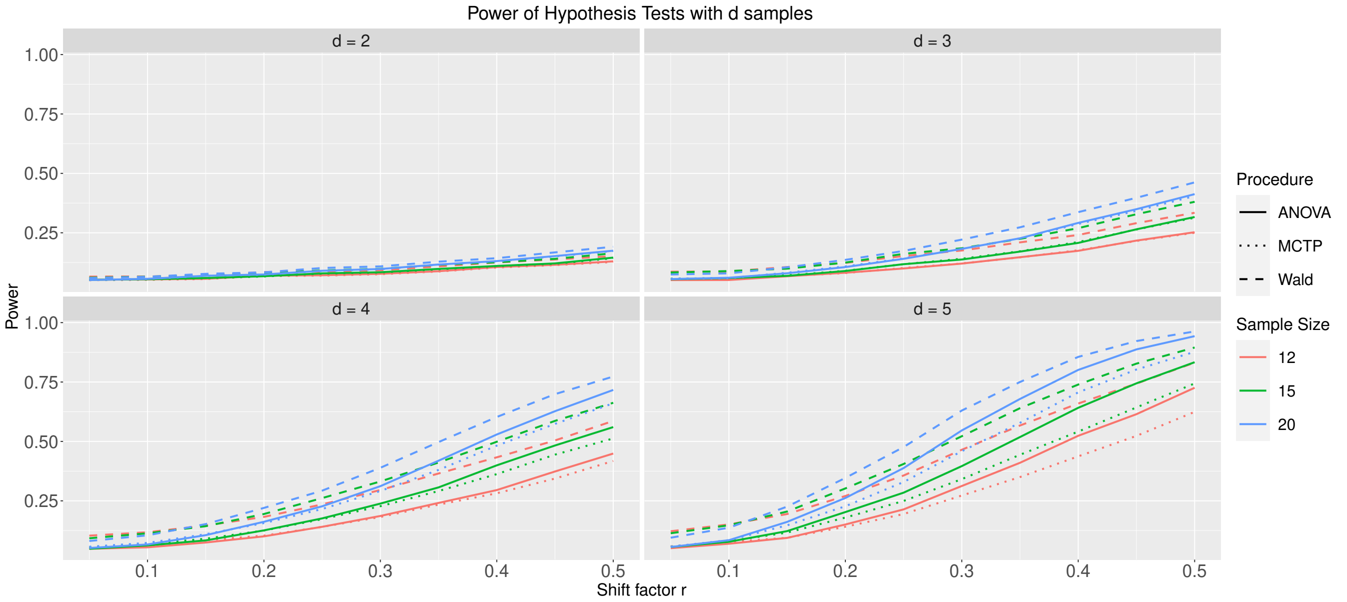

FIGURE B.2.6 Type-II Error Simulation Results for Model 3.3; n_i = 12 (red), 15 (green), 20 (blue); Thick Line: ANOVA-type, Dashed Line: Wald-type, Dotted Line: MCTP; Nominal significance level: α = 0.05

REFERENCES

1. Popoviciu T. Sur les équations algébriques ayant toutes leurs racines réelles. *Mathematica (Cluj)* 1935; 9: 129 - 145.
